# Evaluation of IEEE802.15.4g for Environmental Observations

**DOI:** 10.3390/s18103468

**Published:** 2018-10-15

**Authors:** Jonathan Muñoz, Tengfei Chang, Xavier Vilajosana, Thomas Watteyne

**Affiliations:** 1 Inria, EVA Team, 75012 Paris, France; tengfei.chang@inria.fr (T.C.); thomas.watteyne@inria.fr (T.W.); 2 Computer Science, Telecommunications and Multimedia Department, Universitat Oberta de Catalunya, 08018 Barcelona, Spain; xvilajosana@uoc.edu

**Keywords:** wireless sensor networks, environmental observations, IEEE802.15.4g, sub-GHz, range measurements

## Abstract

IEEE802.15.4g is a low-power wireless standard initially designed for Smart Utility Networks, i.e., for connecting smart meters. IEEE802.15.4g operates at sub-GHz frequencies to offer 2–3× longer communication range compared to its 2.4 GHz counterpart. Although the standard offers 3 PHYs (Frequncy Shift Keying, Orthogonal Frequency Division Multiplexing and Offset-Quadrature Phase Shift Keying) with numerous configurations, 2-FSK at 50 kbps is the mandatory and most prevalent radio setting used. This article looks at whether IEEE802.15.4g can be used to provide connectivity for outdoor deployments. We conduct range measurements using the totality of the standard (all modulations with all further parametrization) in the 863–870 MHz band, within four scenarios which we believe cover most low-power wireless outdoor applications: line of sight, smart agriculture, urban canyon, and smart metering. We show that there are radio settings that outperform the “2-FSK at 50 kbps” base setting in terms of range, throughput and reliability. Results show that highly reliable communications with data rates up to 800 kbps can be achieved in urban environments at 540 m between nodes, and the longest useful radio link is obtained at 779 m. We discuss how IEEE802.15.4g can be used for outdoor operation, and reduce the number of repeater nodes that need to be placed compared to a 2.4 GHz solution.

## 1. Introduction

Low-power wireless mesh (sensor) networks drastically decrease the cost of implementing monitoring/control systems, enabling a wide range of applications in the industrial, environmental and urban context. Wireless mesh networks are used over a wide spectrum of environmental observation applications, including smart agriculture [1], fire monitoring [2], seismic activity logging [3], and snow-pack monitoring [4].

In these applications and many others, the IEEE802.15.4 standard is often used to provide connectivity between sensor nodes. Most deployments use the 2.4 GHz version of this standard, with O-QPSK modulation, 16 frequencies, and a maximum frame length of 127 B [5]. When sensors need to cover a large area, repeater nodes often need to be added to ensure that the deployment is dense enough. Malek et al. [4] for example reported the need to install three times more repeaters than sensor nodes, increasing the cost and time of deployment. With IEEE802.15.4 at 2.4 GHz, radio links are typically 100 m, sometimes up to 300 m, but with a range that drastically decreases when obstacles (such as trees) are between the nodes.

Analysis of sub-GHz-based communications for environmental observations have also been carried out. Kjeldsen et al. [6] and Zhou et al. [7] study the radio waves propagation in sub-terrain environments such as mines and sewers. Both studies conclude that in those environments the radio waves propagate as inside a waveguide. The smaller the cross-section area of the structure, more attenuation happens for lower frequencies (sub-GHz) than for higher frequencies (2.4 and 5.8 GHz). Angles et al. [8] proposed a propagation model for low-power wireless networks in the 868 MHz band, for ground to ground and ground to air communications. The model is validated through experiments. The authors show the decay of the receiver power when the Fresnel radius is obstructed.

Thelen et al. [9] present an extensive set of measurements taken in a potato field of 154 m × 105 m in order to study how the environmental conditions affects the performance of the radio communications. For their experiment they used 13 Mica2Dot nodes with a Chipcon CC1000 radio chip in the 433 MHz band, working with a Frequency Shift Keying (FSK) modulation, 19.2 kbps and +10 dBm TX power. Nodes are placed on the ground and the antennas are at a height of 11 cm. Meanwhile one node transmitted, the rest of the nodes listen and stored the amount of received packets and their Received Strength Signal Indicator (RSSI) value. It is perceived that radio waves propagated better with conditions of high humidity, e.g., during rain and at nights. They concluded that for reliable communication (RSSI ≥−90 dBm), nodes need to be separated for at most 10 m for during blooming (potato plants grow up to 1 m approximately) and 23 m when the crop is on its return.

Hartung et al. [2] described FireWxNet: a hybrid wireless system to monitor weather conditions. This system allows the fire fighters to measure fire and weather conditions in order to predict fire behavior and reduced the damages caused by these events. Two sensor networks were deployed in the Bitterroot National Forest. The nodes used are based on the Mica2 platform featuring a Chipcon CC1000 radio operating at 900 MHz, with a maximum TX power of +10 dBm. Nodes were mounted on a tripod at 1.5 m from the ground and are located on the surface of the Hell’s Half Acre and Kit Carson mountains, at different heights. Stable radio links up to 400 m are formed.

Lazarescu [10] described the design considerations of a low cost WSN for wildfire monitoring. It is used a 50 node deployment using the 433 MHz frequency band, connecting to a gateway embedded inside a wooden birdhouse with solar panels on the top. The antenna of the gateway was left inside the box. This choice reduced drastically the quality of the radio links between gateway and sensor nodes, allowing communication ranges only up to 70 m.

Cerpa et al. [11] presented SCALE, a measurement visualization tool that allow the collection of packet delivery statistics and can help to engineers to determine data capacity and latency of the system. They used the same micro-controller with two types of radios, an RFM TR1000 and a Chipcon CC1000. The former working at 916 MHz, 13.3 kbps with an Amplitude Shift Keying (ASK) modulation and the latter at 433 MHz, 19.2 kbps and FSK. They show that radio links of 50 m with Packet Delivery Ratio (PDR) over 60% are achieved in outdoor environments, with a 0 dBm TX power with the TR1000 radio. Due to the lack of more hardware, they could not test over longer distances. They conclude that there is no evident correlation between PDR and distance for more than 50% communication range.

The IEEE802.15.4 standard continuously evolves. In 2012, the “g” amendment to the standard [12] added a new physical layer, with three modulations (FSK, OFDM and O-QPSK), and a maximum frame size of 2047 B. Most importantly, IEEE802.15.4g is designed to operate in sub-GHz bands (such as 863–870 MHz in Europe, 902–928 MHz band in the US), increasing communication range for the output power, when compared to 2.4 GHz. Nonetheless, it can also be used at 2.4 GHz.

The Wi-Smart Utility Network (SUN) industrial alliance (www.wi-sun.org) has adopted this amendment, specifically the 2–FSK 50 kbps option. This is one of the most frequently implemented options in smart metering applications (other sub-GHz solutions are popular, such as wireless M-Bus, LoRa, Sigfox.). As a result, most “IEEE802.15.4g compliant” chips only implement that option.

In opposition to the 2.4 GHz band, license-free sub-GHz bands are at different frequencies in different countries, and are subjected to regional regulations. This means that devices compliant in one region may not be suitable to be deployed in another. One example is the European regulation, that imposes a duty cycle of <0.1%, or 3.6 s of transmit time over one hour while The United States imposes a duty cycle of <2%.

This study explores the entire “g” amendment, i.e., all three modulations techniques with their further parametrization. The result is that IEEE802.15.4g contains 31 different radio settings, with data rates ranging from 6.25 kbps to 800 kbps, and channel bandwidth from 200 kHz to 1.2 MHz in the European sub-GHz band.

Some studies of IEEE802.15.4g already exist. Sum et al. [13] evaluate the performance of a WPAN when using the IEEE802.15.4/4e/4g for SUNs through simulations. Dias et al. [14] deploy a WSN in office/canteen/warehouse buildings in a smart grid application, and evaluate the performance of a single radio setting (O-QPSK, 250 kbps, 2 MHz channel bandwidth). Mochizuki et al. [15] propose an enhancement to a conventional Wi-SUN system by increasing the transmission power of the downlink communication, and using a 2-FSK 100 kbps setting for their system tested in the city of Kyoto.

Sum et al. [16] study the communication and interference range for deployments for IEEE802.15.4g SUN devices in low and high dense environments (from 10 up to 2500 devices per square kilometer). Based on realistic channel models and assuming FSK modulation with −90 dBm sensitivity, they conclude that the communication range in urban environments (PER ≤ 1%) for SUN devices is 33 m, 65 m and 104 m when using 2.4 GHz, 915 MHz and 460 MHz frequency bands respectively.

Bragg et al. [17] deployed six sensor nodes and two dedicated routing nodes in the Cairngorm Mountains in Scotland. The Zolertia Z1 sensor mote with a CC1120 radiochip was the hardware choice for the nodes. These were separated into two clusters covering one kilometer square area. The border router was located 3.5 km away of the closest router. The data rate was set to 50 kbps. The deployment showed that the radios can provide a single hop communication over that distance. By using a Line of Sight propagation model for a CC2420 radio (implementing O-QPSK PHY, 250 kbps at 2.4 GHz), they determined that for a solution using this radio would need at least 25 routing nodes.

Through simulations and outdoor experimentation, Kojima et al. [18] investigate the feasibility of employing the Gaussian FSK PHY under multi-path conditions in a suburban area. With transmitter and receiver tuned at 413 MHz, with a BPSK modulation and a TX power of +10 dBm, the radio signal is received with a power level of −60 dBm. Results show that for a frame length of 1500 B, PER ≤ 10% and considering a receiver sensitivity of −100 dBm, the coverage area of the radio link is a few hundred meters.

All these IEEE802.15.4g studies and experiments have something in common: they only consider a single radio setting.

What is missing is a comparative performance evaluation of all possible radio settings of IEEE802.15.4g, with a particular focus on communication range, performance and limits for different scenarios. Through experimentation, we test all IEEE802.15.4g radio settings in different scenarios where a wide range of applications can take place.

The contribution of this article is three-fold:

1. We define a system to carry out range testing of different radio settings with automatic out-of-band synchronization and minimum human intervention.
2. We determine the best radio setting(s) to be used in the scenarios covered in this study, taking into account PDR, “goodput” (nominal data rate times the PDR), electric charge consumption and communication range.
3. We provide an executive summary of the regulation on sub-GHz band usage in Europe, the US and Japan.

The remainder of the article is organized as follows. Section 2 details the materials used for the experiments, explains the main sub-GHz regulation, and describes the characteristics of the scenarios and PHYs tested. Section 3 shows the PDR for each scenario at different communication ranges. Section 4 determines the most fitting PHYs per scenario according to PDR value, throughput and power consumption. Section 5 concludes this article by presenting future work.

## 2. Materials and Methods

We use the networking term “PHY” (physical layer) interchangeably with “radio setting”.

Section 2.1 provides an overview of the PHYs being tested, Section 2.2 gives the main characteristics of the regulations of Europe, the United States and Japan when using sub-GHz bands. Section 2.3 and Section 2.4 describe the hardware and software used in these range test experiments, respectively. Section 2.5 details the scenarios where the nodes were deployed.

### 2.1. IEEE802.15.4g PHYs

The “g” amendment of the IEEE802.15.4 standard has been specifically designed to target SUN applications. Its maximum packet size is 2047 B, enough to carry a complete IP packet in one frame without the need to implement link-layer fragmentation. A longer communication range is needed by SUN applications, therefore the standard makes a more extensive use of the sub-GHz frequencies than the previous version of 2006, using O-QPSK.

In sub-GHz bands, radio signals can cover longer distances with the same transmission power, compared to 2.4 GHz.

In Europe, two of the most popular unlicensed ISM sub-GHz frequency bands for Non-Specific Short Range Devices are 433.05–434.79 MHz and 863–870 MHz [19,20]. For this study, we use the latter because the frequency band is wider (7 MHz vs. 1.74 MHz), allowing more communication channels. This band also provides a good trade-off between range and antenna dimension (the recommended size of an antenna depends of the wavelength—λ—of the signal. For dipole antennas, using length λ/2 is common. At 433 MHz, this translates to a 35 cm antenna, compared to a 17 cm at 863–870 MHz).

The IEEE802.15.4g includes three modulations:

- **Frequency Shift Keying (FSK).** In transmission, it delivers good power efficiency thanks to the constant envelope of the signal. It may implement a Gaussian filter or be unfiltered. It uses convolutional coding as Forward Error Correction (FEC). This technique uses redundancy of information and gives the possibility to the receiver to correct errors by itself, without having to request a retransmission of the information. The price to pay is the reduced effective data rate, since more bits are needed to encode the same information. A FEC rate of 1/2 means that for 1 bit of information, 2 bits are transmitted. Data Whitening and interleaving are also optional. Data rates vary from 5 kbps to 400 kbps. SUN FSK devices are simple and do not require complex circuitry consuming high processing power, resulting in a less power-hungry technology. This PHY targets low data rates and high energy efficiency applications, e.g., smart metering. Most electric meters in the US use this PHY.
- **Orthogonal Frequency Division Multiplexing (OFDM)**: This PHY is designed to provide high data rate communication in challenging environments presenting multi-path fading conditions, such as urbanized areas or indoor scenarios. Here, the transmitted signals bounce off obstacles, taking different paths and some of them arriving to the receiver at slightly different times (with a different phase). As a result, the overall received signal is the complex sum of those reflections, which can constructively or destructively (self-)interfere. Multi-path fading is also dependent on the frequency used, i.e., it affects different frequencies in different ways. In order to cope with this, OFDM spreads the information to be transmitted over multiple sub-carriers occupying different frequencies. Each sub-carrier is modulated according to a Modulation and Coding Scheme (MCS) value, and it can be BPSK, QPSK and 16-QAM. If part of the data is lost, it can be recovered using FEC techniques and frequency repetition (2 or more sub-carriers transporting the same information). OFDM devices require a more complex circuitry with higher power consumption in order to carry out sophisticated signal processing functions such as creating or demodulating OFDM symbols. This technology has been widely applied in high-end systems such as cellular networks; its use in low-power devices is new. Data rates vary from 50 kbps up to 800 kbps. Typical applications are security and surveillance systems, where high data rates are required.
- **Offset Quadrature Phase Shift Keying (O-QPSK).** It uses Direct Sequence Spread Spectrum (DSSS) or Multiplexed Direct Sequence Spread Spectrum (MDSSS), with data rates from 6 kbps up to 500 kbps. It shares some characteristics with IEEE802.15.4-2006, making multi-mode systems easier to design and more cost-effective [21].

These PHYs accept further parametrization, either by modifying the data rate (more information per symbol transmitted or more symbols per unit of time) and/or channel bandwidth. In this study, each PHY with a specific set of parameters is defined as radio setting and identified by an “alias”. In the 863–870 MHz frequency band, a total of 31 radio settings have been tested.

Table 1 describes the radio settings for all the combinations of parameters using FSK and provides an “alias” to each of them. Table 2 and Table 3 provide the same information for OFDM and O-QPSK, respectively. Only for the FSK radio settings implementing $\frac{1}{2}$ rate FEC, the actual data rate is halved, since every two bits sent by the radio correspond to 1 bit of information. For the rest of the radio settings, the data rates stated in Table 1, Table 2 and Table 3 are the data rates of the actual information leaving the radio.

Using sub-GHz bands has two main drawbacks. First, license-free sub-GHz bands are not at the same frequency in all countries—unlike the 2.4 GHz ISM band—and each country/region makes its own selection of frequency to be assigned to unlicensed users:

- In Europe, 863–870 MHz, with upcoming availabilities in the 870–876 MHz (early stages).
- In the United States, 902–928 MHz.
- In Japan, 922.4–928 MHz.

Second, each country/region imposes (different) regulations to these sub-GHz bands. This significantly reduces the throughput of these networks, and products for one country may not satisfy the regulations of another.

### 2.2. Regional Regulations

Duty cycle, maximum bandwidth per communication channel, maximum dwell time and maximum transmission power are some of the parameters each region/country regulates. This section summarizes the regulations in Europe, the United States and Japan.

#### 2.2.1. Europe

The European Telecommunication Standards Institute (ETSI) and the Electronic Communications Committee (part of the European Conference of Postal and Telecommunications Administrations, CEPT) define the set of recommendations and standards [19,23] for Short Range Devices operating in the 25–1000 MHz frequency band.

Considering that the CEPT includes 48 European countries (national level) and ETSI has more than 800 members (regulatory bodies, governments, companies, universities, research bodies) from 66 countries and five continents, their normative and regulations are complex, as consensus within the parties have to be made. In addition, some countries may differ on the designated bands or impose certain conditions on uses of specific bands.

The normative for EU wide harmonized National Radio Interfaces cuts the 863–870 MHz band into sub-bands, as detailed in Table 4. Table 5 shows conformance to any National Radio Interface, in the case of using the entire 7 MHz band.

The duty cycle restriction of 0.1% in some of the sub-bands could be eased and taken up to 2.8% if polite spectrum access is applied to the transmitters. Polite spectrum access refers to Listen Before Talk (LBT) and Adaptability Frequency Agility (AFA). Prior to transmission, a “polite” device performs Clear Channel Assessment (CCA) to check its availability. If the channel is free, the device continues its transmission. If it is not, the device should not retry its transmission until a random time has passed. Optionally, the device can change the intended transmitting frequency and listens again before starts the transmission.

The timing parameters of polite spectrum access are:

- Minimum CCA time: 160μs
- Maximum single TX duration: 1 s
- Maximum cumulative TX time in one hour: 100 s (duty cycle of 2.8%) per 200 kHz spectrum

#### 2.2.2. United States

The Federal Communications Commission is the body in charge of the regulation of the radio electric space in the US [24]. For the 902–928 MHz ISM band, its use is limited to frequency hopping and digitally modulated radiators. US duty cycle regulation is more permissive, as well as the maximum TX power, than the European regulation.

In the case of frequency hopping systems, devices have to be compliant with:

- Channel hopping carrier frequencies should be separated by the greater between 25 kHz or the 20 dB bandwidth channel.
- If the 20 dB bandwidth of the hopping channel is <250 kHz, at least 50 hopping frequencies should be used and each up to 0.4 s per 20 s period.
- If the 20 dB bandwidth of the hopping channel is ≥250 kHz up to 500 kHz, at least 25 hopping frequencies should be used and each up to 0.4 s per 10 s period.

For the case of systems using digital modulation techniques, the minimum 6 dB bandwidth should be at least 500 kHz.

The maximum peak output power is:

- If using channel hopping: 30 dBm if at least 50 hopping channels are used, 21 dBm if less than 50 hopping channels are used (minimum 25 hopping channels).
- If using digital modulation: 30 dBm. A duty cycle of 0.4 s each 20 s gives us a channel occupancy of 2% and in the best case, 0.4 s each 10 s, 4%.

#### 2.2.3. Japan

The Association of Radio Industries and Businesses defines the use of the 922.4–928 MHz band [25], using carrier sense under these premises:

- Minimum listening time during CCA of 128μs; maximum: 5 ms.
- Maximum single TX time: 400 ms.
- Duty cycle ≤ 10%.
- If the previous TX time is >200 ms, the device shall wait for at least 10 times the TX time before the next TX.
- If the previous TX time is ≤200 ms and more than 6 ms, it shall wait for 2 ms before consecutive TX.
- Using two radio channels at the same time (i.e., signal is 400 kHz wide), the maximum single TX time has to be less than 200 ms.
- Using up to 5 radio channels at the same time, maximum single TX time has to be less than 100 ms.
- Maximum TX power is 20 mW.

If no carrier sense is used:

- Maximum TX power is 1 mW.
- Maximum single TX time: 100 ms.
- Duty cycle ≤ 0.1%.

### 2.3. Hardware

This section describes the hardware used in the range measurement campaign. We used 4 nodes, 1 configured as a TX (transmitter) and 3 others as RX (receiver). Each node is mounted on a 1.8 m PVC tube. From Watteyne et al. [1] and Malek et al. [4], we see their choice of putting their sensors at a height of 4 m on top of fixed wooden poles, reducing the impact the ground poses as an obstacle to the radio links. In our case, 1.8 m tubes reach the same height at which smart meters are located in a house. Therefore, the impact of the ground being an obstacle (e.g., Fresnel Zone obstruction) is a condition nodes have to cope with.

#### Node

**Figure 1:**
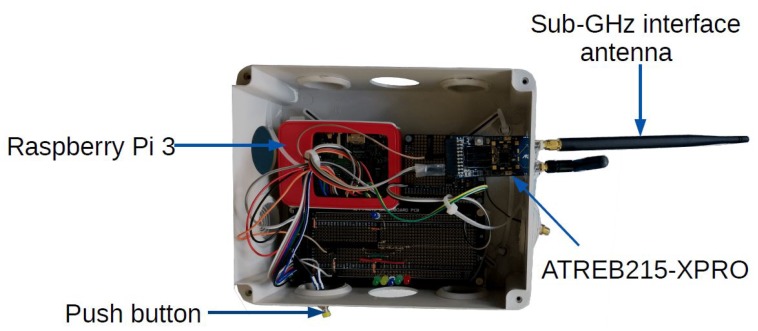
Node used in the range test measurement campaign.

A node consists of a Raspberry Pi 3 (rPi) model, an ATREB215-XPRO-A radio board evaluation kit, a 2 dBi omni-directional antenna, a GPS module and a push button; all housed in a plastic box. The rPi controls the radio board through a SPI bus. The electronic devices are powered by a 22,000 mAh battery bank. Figure 1 shows a node.

**ATREB215-XPRO-A:** This radio board features an AT86RF215 radio chip implementing both the IEEE802.15.4-2006 and the IEEE802.15.4g standards. It contains two transceivers, one for sub-GHz frequencies and the other for the 2.45 GHz frequency band. It has two SMA connectors, one for each transceiver, where we connect 2 dBi 1/2 wavelength whip antennas. It is driven through an SPI bus. It is powered by +3 V from the rPi. Table 6 shows the sensitivity for each radio setting in the sub-GHz transceiver.

**Raspberry Pi:** Each rPi has a Linux Debian distribution. Through a SPI bus, it drives the radio board, configuring it on each radio setting to be tested. After each range test experiment, it stores the results of each test to a system file.

**GPS module:** We used the Ultimate GPS module from Adafruit, with an external uFL connector to an active GPS antenna. This module is built around the MTK3339 GPS chip-set. It provides the rPi with the Greenwich Mean Time (GMT) and the position. Each rPi has the same System time, making them tightly synchronized.

### 2.4. Software

**Figure 2:**
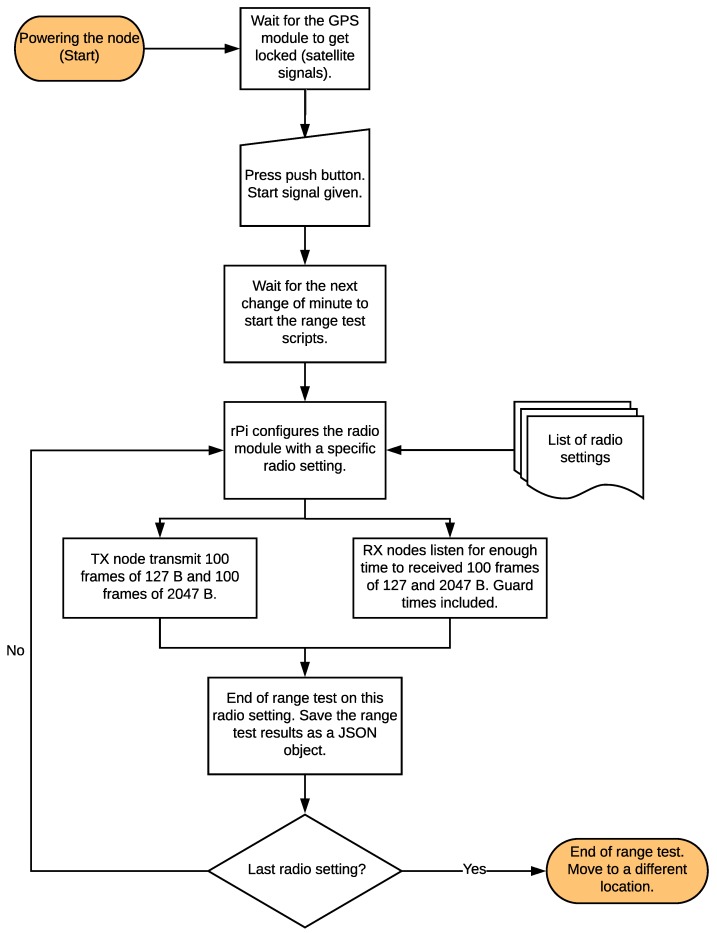
Diagram of the steps followed by each node during an experiment.

The range test scripts are written in Python and they perform the processes depicted in Figure 2. When powering the nodes, the GPS modules at each node wait for satellite signals. Once the GPS gets a lock signal, they feed the rPis with GMT and position, enabling the experiment scripts to start. The test script then waits for the signal from the push button to start the experiment, therefore a person per node is needed. When the signal is received from the push button, the script waits for the start of the next minute. They drive the radio board through an experiment, making the TX node loop over the 31 radio settings and sending burst of 100 frames of 127 B and 100 frames of 2047 B for a total of 200 frames sent per radio setting. On the RX nodes, the script configures the radio board with the same PHY and frequency as the TX node at the same time. Since the System time at the nodes is the same (GMT, fed by the GPS module), the nodes are tightly synchronized.

An experiment consists in the node sending 100 frames of 127 B and 100 frames of 2047 B, using the 31 radio settings shown in Table 6. 127 B is the maximum size of the previous version of the standard, defined in 2006, that uses O-QPSK with 250 kbps, and that can be used just as a comparison for the interested reader willing to perform the same experiments with that technology. 2047 B is the maximum size of the ‘g’ amendment, and now appended to the standard as SUN-PHYs. We measure the time the TX node takes to transmit 100 frames of 127 B and 100 frames of 2047 B over each one of the radio settings tested, therefore we know for how long RX nodes need to listen on every configuration. Appropriate guard times are taken into account, in order to guarantee that the RX nodes are listening when the TX node starts transmitting. With this information, we build the sequence of radio settings that the nodes need to implement and for how long. Since the nodes start executing the range test scripts aligned with the next change of minute, they are synchronized within milliseconds.

The inter-frame spacing time is 20 ms, enough to avoid collisions between consecutive frames. Each frame has a sequence number, allowing us to know which frames were well received and which were not. The frames sent in the range test experiment do not have any MAC or Network addresses. They are filled with dummy data with just a 2 B sequence number and a 4 B Frame Check Sequence (FCS), checking the correctness of the frame in the RX side.

RX nodes log, for each frame received, the radio setting and frequency it listens on, the RSSI value and the correctness of the FCS. This information is stored as a JSON object in a file system, in addition to the GPS information (position and time). Because 100 frames are sent on each radio setting and length, the PDR can be computed (as an online addition to this article, the test scripts and documentation is available at https://github.com/openwsn-berkeley/range_test).

### 2.5. Scenarios

**Figure 3:**
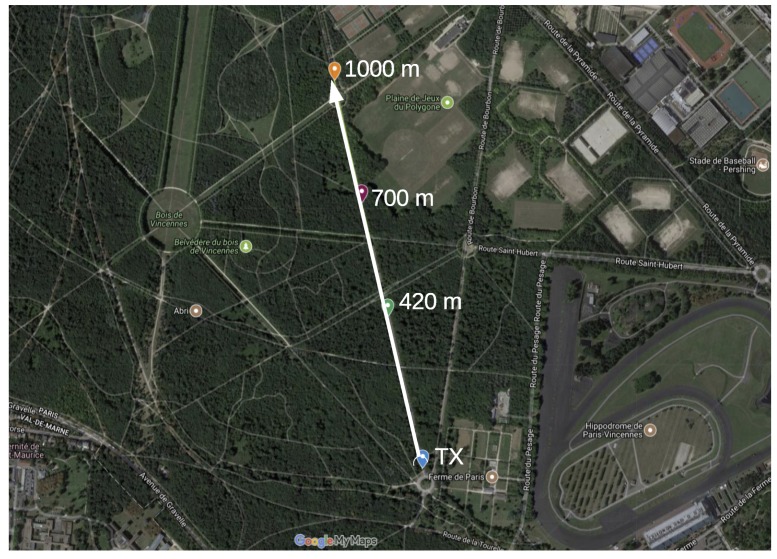
LoS scenario. Distances show where the RX nodes are placed away from the TX node.

**Figure 4:**
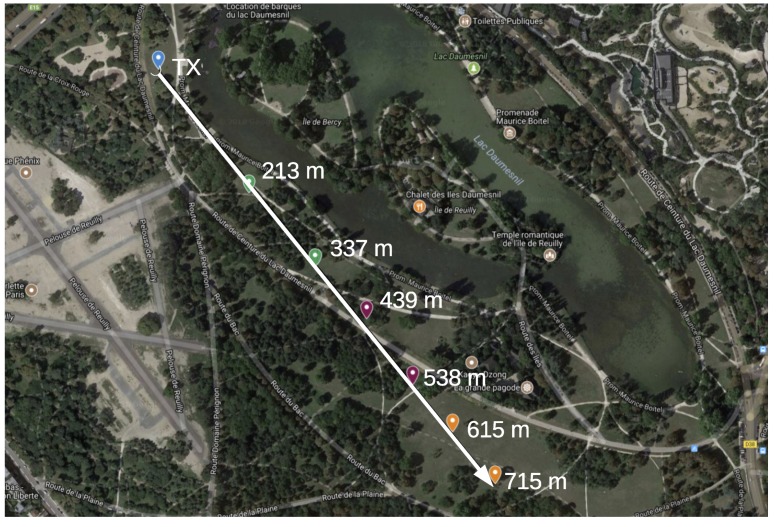
Smart Agriculture scenario.

**Figure 5:**
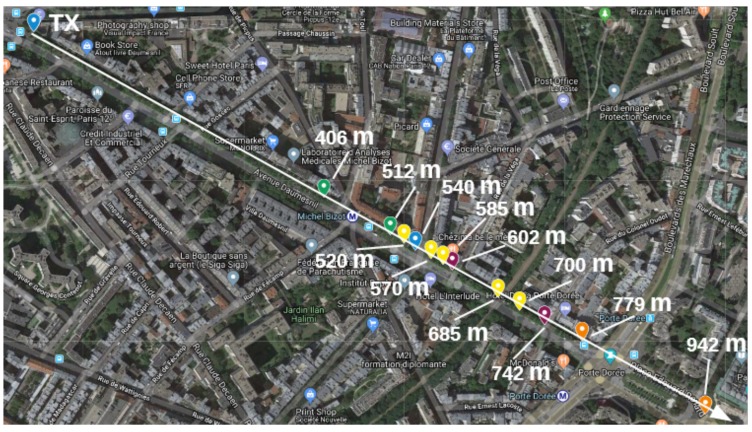
Urban Canyon scenario.

**Figure 6:**
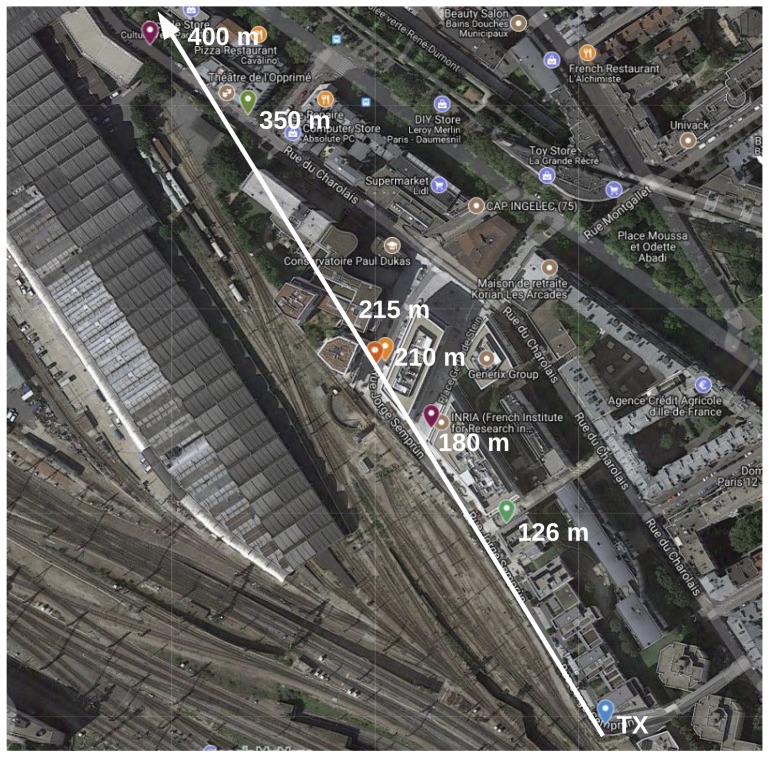
Advance Metering Infrastructure scenario.

The range test experiments are carried out in the city of Paris, France, in 4 scenarios, each being a likely IoT application environment. These are:

- **Line of Sight (LoS)**: nodes are deployed in the *Bois de Vincennes*, on a pedestrian 12 m wide asphalted route (*Rue Dauphine*). The area is characterized by dense vegetation with tall trees at both sides of the route. No important obstruction is between the TX and RX nodes during the length of the experiment with people occasionally crossing this path. Numerous IoT applications are foreseen in this scenario: monitoring natural resources on a prairie-like environment, smart metering in the country side, smart grid in rural areas, livestock monitoring, mining and more. Figure 3 depicts the location where the nodes are deployed, and the distances between them.
- **Smart Agriculture**: nodes are deployed in the Parc de Vincennes, next to the Lac Daumesnil. There are trees between the TX and RX nodes, obstructing the direct path between the nodes. This scenario mimics IoT application environments such as: Smart agriculture, monitoring natural resources on a forest-like environment, livestock monitoring on a vegetation-abundant terrain and more. Figure 4 shows the deployment setup and the distances between the nodes.
- **Urban canyon**: we chose the Avenue Daumesnil, a 35 m wide urban canyon. This is a busy environment, with people walking and automobiles transiting across and along the path between the nodes. There are buildings on both sides of the street (up to 10 stories), and with trees along the avenue. This scenario is representative of some Smart City applications, including parking, metering, lighting, traffic control, pollution monitoring, etc. Figure 5 depicts the nodes’ locations and the distance between them.
- **Advanced Metering Infrastructure (AMI)**: nodes are located along the street Jorge Senprùm, next to the Inria buildings in Paris. The TX node is located at one extreme of a small neighborhood; the RX nodes are positioned between buildings, with no LoS to the TX node. Urban Advanced Metering applications are deployed in this type of scenario. Figure 6 details the position of the nodes and the distance between them.

## 3. Results

This section provides the results as follows. We show the PDR value for every radio setting of every node on every scenario previously described. We use the terms useful PDR and high PDR for values of at least 50% and 75% respectively.

### 3.1. Line of Sight (LoS)

RX nodes are located at 420 m, 700 m and at 1000 m from the TX node. Table 7 shows the PDR value for the three RX nodes considering packets of 127 B and 2047 B. At 420 m, radio settings with high data rate have a PDR around 100%. At 700 m, the PDR stays between 70% and 100% for radio settings using FSK and O-QPSK. Radio settings using OFDM have a poor PDR, in most cases incapable of getting any frame across. The exception is OFDM2-100, with PDR of 92% for short frames and 47% for long frames.

The poor performance of OFDM at 700 m, in comparison to FSK and O-QPSK PHYs is due to that the TX power is not the same for all radio settings. According to Table 6, the maximum TX power for OFDM radio settings is +11 dBm whereas for the rest is +14 dBm. The robustness of OFDM can be shown in strong multi-path environments (e.g., dense cities), where singled-carrier radio signals would suffer from self-Interference caused by multiple rays of the same symbol arriving at different time and interfering with subsequent symbols. Here it is not the case. We see how singled-carrier signals have a good PDR and OFDM PHYs do not. Therefore, the low performance of OFDM is due to the attenuation the radio signal suffers through the path towards the RX node.

At 1000 m, the radio link is almost nonexistent, not useful for data exchange.

### 3.2. Smart Agriculture Scenario

In this scenario, the experiment is run twice during the same day and with equal weather conditions. RX nodes are located at 213 m, 439 m and 615 m from the TX node on the first run and at 337 m, 538 m and 715 m on the second run. Table 8 shows the PDR for all the RX nodes, for packets of 127 B and 2047 B. High data rates radio setting with high PDR are achievable at least up to 337 m. At 615 m, the radio link allows communication of at least 50 kbps with high PDR value. At 715 m, only OQPSK-12.5 present a high PDR for short packets.

### 3.3. Urban Canyon

In this scenario we collect measurements from 12 locations, between 406 m and 942 m and within 3 non-consecutive days with similar conditions (sunny days, at noon). Table 9 and Table 10 show the PDR for packets of 127 B and 2047 B on each RX node location.

In this scenario, interference and multi-path fading are expected since this is a high populated area with many buildings along the street, with smart metering devices already implemented and other applications accessing the same frequency band. As shown in Table 9, the PDR for OFDM2 is lower at 406 m than at 512 m. An explanation for this is external interference. These two measurements were not taken at the same moment, and we can also see that the interference was present during the transmission of packets with OFDM2-50, OFDM2-100, OFDM2-200 and OFDM2-400. Before and after that time, PDR rose to values close to 100%. In addition, for the following node locations, these high values are maintained.

After 540 m, there is a negative slope in the street level and after 685 m there is a viaduct which is perpendicular to the avenue Daumesnil. High data rates with high PDR are achieved at least up to 540 m, and the maximum coverage of the radio link is around 780 m.

### 3.4. Advanced Metering Infrastructure

In this scenario, we run the experiment twice during non-consecutive days having similar weather conditions. In the first run, RX nodes are located at 126 m, 180 m and 215 m. In the second run, RX nodes are located at 210 m, 350 m and 400 m. All nodes located within 215 m, even without line of sight, have a PDR close to 100% for almost all radio settings considering packets of 127 B and 2047 B. There are some buildings between the TX node and the RX nodes located at 350 m and 400 m. This severely affects the quality of the radio link. In these two node locations only the O-QPSK radio settings and in less proportion the 2FSK-FEC-50 are able to get connectivity. These PHYs happen to be the most sensitive of the radio. Table 11 shows these PDR values for all the RX nodes.

## 4. Analysis

In this section, we provide an analysis per scenario based on three parameters: PDR, throughput and electric charge consumption (the reason we provide the electric charge (C) consumption is that low-power devices are usually battery-powered and the voltage supply is considered fixed. We acknowledge the fact that electric circuitry consumes electric energy (J) and not only electric charge (C)).

The first parameter, PDR, tackles latency. Making the assumption that retransmissions do not take place right after the failure of a packet exchange, this time between retries increases latency. Also, a radio link with high PDR is of utmost interest in order to reduce retransmissions and thus, electric charge consumption.

The second parameter, throughput, takes into account the nominal data rate of each radio setting times its PDR. The result is the “goodput”, and with this value we can calculate the maximum amount of packets that can be exchanged during a given time.

European regulation requires the use of this frequency band with a duty cycle < 0.1%. Accordingly, we calculated the maximum amount of correct packets we could send during one hour, which corresponds to 3.6 s combined transmission time. Equation (1) shows how we calculated this number.

(1) $$\mathrm{Max}\_\mathrm{Packets}\leq \frac{3.6\;s\times \mathrm{DR}(B/s)\times \mathrm{PDR}}{\mathrm{Packet}\;\mathrm{size}(B)+\mathrm{Packet}\;\mathrm{overhead}(B)}$$

where 3.6 s is the maximum time a node can transmit per hour and DR is the nominal data rate of the radio setting. Packet size is the length of the data in the PHY (PSDU), and Packet overhead includes the PHY header and the Synchronization Header (in the case of FSK and O-QPSK) (the Synchronization Header is composed of a Preamble and a Start of Frame Delimiter (SFD). We consider the shortest preamble defined in the standard [22] for our calculation.) or the Short Training Field (STF) and Long Training Field (LTF) (in the case of OFDM). The addition of Packet size and Packet overhead result in the total amount of bytes the radio needs to transmit in order to send a frame.

The third parameter, electric charge consumption, is obtained by calculating how much electric charge is needed to transmit a single packet. We take into account all radio activity, including the packet overhead and packet size (PSDU). Equation (2) shows how we get the electric charge per packet. The $I_{TX}$ value is the current drawn by the radio module when it is in transmission mode (see Table 6).

(2) $$\mathrm{Electric}\;\mathrm{charge}\;\left(\mathrm{Coulombs}\right)\geq \frac{\mathrm{Packet}\;\mathrm{size}(B)+\mathrm{Packet}\;\mathrm{overhead}(B)}{\mathrm{DR}(B/s)\times \mathrm{PDR}}\times I_{TX}\;\left(A\right)$$

The radios used in the experiment are powered with 3 volts (V). The electric energy (J) the radios consume can be derived from Equation (2) ×3V (Energy(J)=C×V).

We see that the PDR value is present in both (1) and (2). This takes into account the potential number of retransmissions needed in order to get one correct packet at the receiver side.

For each scenario, two node locations are considered: the longer coverage where high data rates can be achieved and the maximum coverage of the radio link, despite of the data rate. For these node locations, we differentiate short (127 B) and long (2047 B) packets.

We take the value of 2FSK–50 as a reference because this is the most used configuration in the industry for smart metering applications worldwide. In addition, this PHY is the one designated to be used as the Common Signaling Mode (CSM) during the Multi-PHY Management procedure defined in the standard [22]. Moreover, the 2FSK-50 radio setting is the mandatory mode for the Wi-SUN alliance in the US and in Europe.

In the following graphs, we highlight the highest values of each parameter with dotted bars and the reference 2FSK–50 with a striped bar. A dotted line is added as a reference on every figure to easily compare the 2FSK–50 kbps radio setting with the rest of the PHYs. Although in most cases 2FSK–50 delivers a good PDR in comparison with most of the other PHYs, this paper shows that there may be more interesting PHYs depending on the constraints of range, throughput, duty cycle and electric charge consumption.

### 4.1. Line of Sight Scenario

High data rates can be achieved up to 420 m, and the maximum length of the radio link with low data rates can reach 700 m with a high PDR. There is not useful radio link at 1000 m.

#### 4.1.1. RX at 420 m

**Figure 7:**
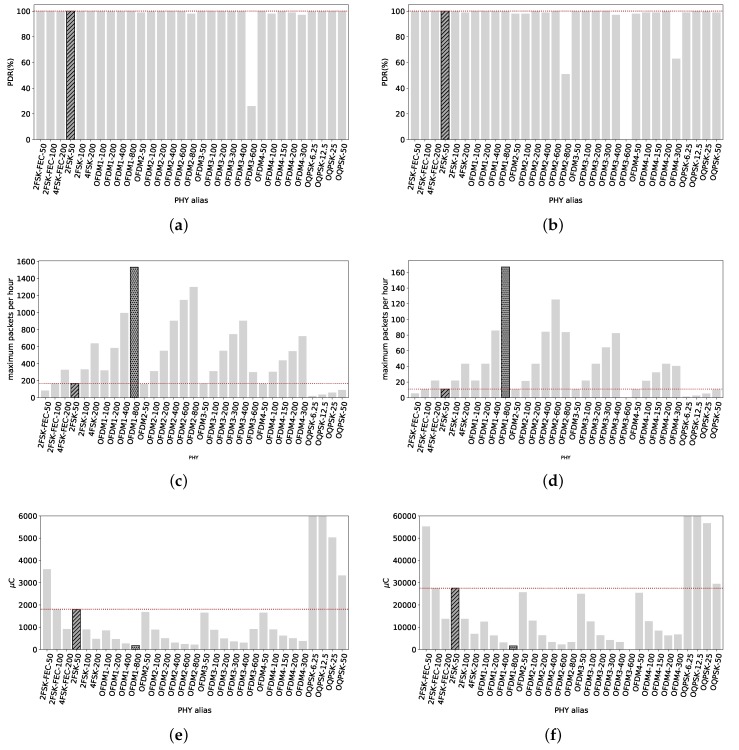
Line of Sight, 420 m. The radio link allows the transmission of packets with 2047 B even with the highest data rates available with a PDR very close to 100%. Within this distance, high data rates radio settings maintain high reliability while consuming less electric charge and allowing more data exchange than low data rates radio settings. (**a**) PDR of all radio settings at 420 m from TX with packets of 127 B. Except for OFDM3-600, all radio settings have a PDR close to 100%; (**b**) PDR of all radio settings at 420 m from TX with packets of 2047 B. OFDM2-800 and OFDM4-300 do not have PDR values close to 100%. OFDM3-600 has 0% PDR; (**c**) Maximum amount of 127 B packets that can be correctly sent per PHY under 0.1% duty cycle regulation. OFDM1-800 can send 1531 packets while 2FSK-50 only 166; (**d**) Maximum amount of 2047 B packets that can be correctly sent per PHY under 0.1% duty cycle regulation. OFDM1-800 can send 167 packets while 2FSK-50 roughly 11; (**e**) Average electric charge consumption per packet of 127 B correctly sent. OFDM1-800 is the most electric charge-efficient, consumes 178.6 μC whereas the reference 2FSK-50 consumes 1807 μC; (**f**) Average electric charge consumption per packet of 2047 B correctly sent. OFDM1-800 consumes the lowest amount of electric charge per packet, with 1637 μC whereas the reference 2FSK-50 consumes 27.5 mC.

As shown in Figure 7a for a packet size of 127 B, most of the radio settings have a PDR close to 100%, including the reference 2FSK-50. OFDM3-600 is the only exception, with a 26% PDR. The majority of the radio settings has a high reliability at 420 m with LoS.

Increasing the packets size to 2047 B, Figure 7b shows that the performance of the radio settings is not much affected, with high data rates still having a PDR close to 100%, including the reference 2FSK-50. The exception in this case are OFDM2-800, OFDM3-600 and OFDM4-300, the highest data rates of OFDM options 2, 3 and 4. One of the reasons is that these PHYs present the lowest sensitivity (the lower the sensitivity value is, the higher sensitivity is. e.g., a device A with sensitivity of −110 dBm and another device B with sensitivity of −120 dBm, device B has a higher sensitivity than device A.) (−101 dBm, −97 dBm and −101 dBm respectively) of the different OFDM radio settings. The poor performance of OFDM3-600 (with a 26% PDR) could be linked to its low sensitivity, being at least 4 dB lower than any other OFDM radio setting. The sensitivity for 4FSK-200 is 1 dB lower but the TX power is 4 dBm higher. Therefore, the signal at the receiver is 4 dB higher for the 4FSK-200 PHY, enough to present a PDR of 100%.

On the other hand, the rest of the PHYs present high reliability. Thus, we do not highlight any particular PHY in Figure 7a,b since most of them have 100% PDR.

Considering throughput, with 127 B packets, Figure 7c clearly shows that OFDM1-800 is the PHY than can have the maximum amount of correct packets transmitted within an hour (duty cycle of 0.1%), with 1531 packets. The reference value, 2FSK–50, can transmit during the same time just 166 packets.

This proportion is maintained with packets of 2047 B, as shown in Figure 7d. OFDM1-800 stands alone with the possibility of transmitting 167 packets against roughly 11 of 2FSK-50 whilst having 0.1% duty cycle. Therefore, OFDM1-800 has the capability of transmitting more short and long packets than any other PHY within the standard, under the 0.1% duty cycle regulation and at 420 m from the TX. If it is the interest of the user to maximize the amount of packets that can be sent, OFDM1-800 or any other OFDM option with high data rate can be used, as Figure 7c,d show.

Figure 7e shows the average electric charge consumption per packet transmitted for all radio settings. We can see that high data rates PHYs, mostly OFDM, consume several times less electric charge that the reference 2FSK-50. The most electric charge-efficient is, again, OFDM1-800. It consumes 178.6 μC to get a 127 B packet across while 2FSK-50 consumes 1807 μC.

For long packets, average electric charge consumption is depicted in Figure 7f. The tendency is maintained, high data rates consumed less electric charge than the rest. 2FSK-50 consumes on average 27.5 mC while OFDM1-800 only 1.637 mC.

#### 4.1.2. RX at 700 m

**Figure 8:**
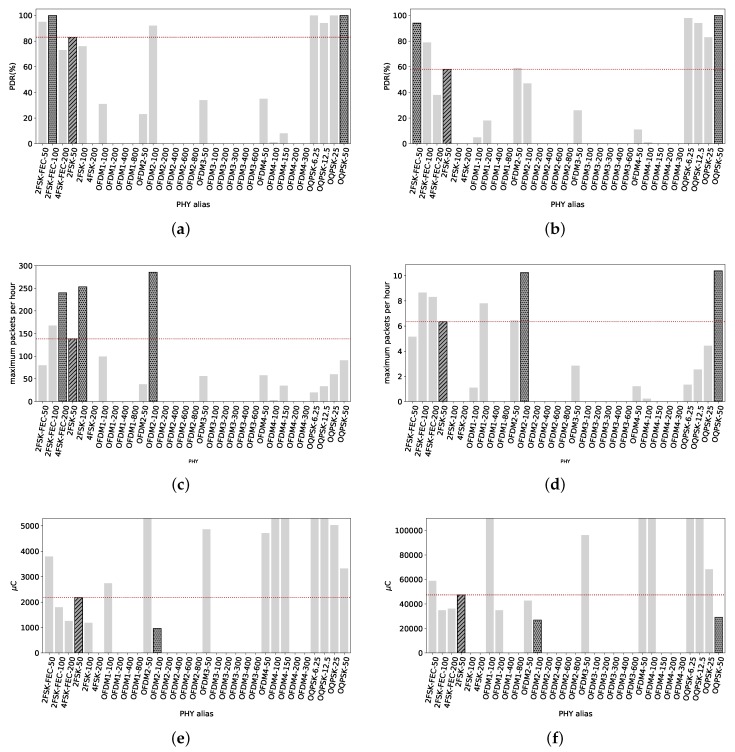
Line of Sight, 700 m. Only O-QPSK and FSK radio settings are able to provide the highest reliability. This node is close to the limits of the coverage. OFDM radio settings have a poor performance at this distance, but the difference is that OFDM transmissions are at least 3 dBm weaker that the rest of the PHYs. (**a**) PDR at 700 m from TX with packets of 127 B. Highest reliability achieved with 2FSK-FEC-100, OQPSK-6.25, OQPSK-12.5 and OQPSK-50. The reference has 83% PDR. OFDM2-100 has also higher reliability than the reference; (**b**) PDR at 700 m from the TX with packets of 2047 B. The reference has 58% PDR, while 2FSK-FEC-50 94% PDR and OQPSK-50 100% PDR; (**c**) Maximum amount of 127 B packets that can be correctly sent within an hour. 4FSK-FEC-200 and 2FSK-100 can send 239 and 253 short packets. OFDM2-100 can send up to 285 packets, while the reference only 138 packets; (**d**) Maximum amount of 2047 B packets that can be correctly sent within an hour. OFDM2-100 and OQPSK-50 can send up to 10 packets, while the reference 2FSK-50 just 6 packets; (**e**) Average electric charge consumption per packet of 127 B. OFDM2-100 consumes the less electric charge, 964 μC while 2FSK-50 consumes 2178 μC; (**f**) Average electric charge consumption per packet of 2047 B. OFDM2-100 and OQPSK-50 are the less electric charge-hungry radio setting, consuming 26.8 mC and 29.1 mC respectively while 2FSK-50 consumes almost the double, 47.5 mC.

Considering the RX node at 700 m, we approach to the limit of the radio link. Figure 8 shows that high data rates are not capable of delivering any packet, making them unusable for similar distances.

From Figure 8a, with short packets, we see that most OFDM radio settings have a poor or nonexistent PDR. The reference 2FSK-50 has a PDR of 83%. Only OFDM2-100 presents a PDR of 92%. O-QPSK radio settings have a high reliability, with 3 out of 4 PDR values of 100% and the remaining (OQPSK-12.5) of 94%. 2FSK-FEC-100 has 100% PDR. Therefore, we highlight 2FSK-FEC-100 and O-QPSK since they have the highest PDR and highest data rate in their technology.

Increasing the packet size to 2047 B, we see from Figure 8b that the PDR slightly drops in comparison to small packets. Nonetheless, it is still around 80% for some radio settings. The reference is at 58% PDR, being matched by OFDM2-50 and outperformed by all O-QPSK PHYs and 2FSK-FEC-50 and 2FSK-FEC-100. We highlight the highest PDR of each technology, therefore 2FSK-FEC-50 and OQPSK-50.

It is notable that for OFDM2-50, the PDR concerning long packets is 59% whereas for short packets is only 23%. This is counterintuitive since short packets should have a higher PDR than long packets due to is lower probability of getting a wrong symbol throughout the complete received frame. Therefore, this effect can be attributed to interference that occurred only in that precise moment. For the following radio setting tested, OFDM2-100, we see how the PDR for short packets increased to 92%, using the same frequency as OFDM2-50, which shows that the interference is no longer present.

Ref. [17] shows that a link of 3.5 km can be obtained with this technology. They were in the Highlands of Scotland and the router node with that link was located at the top of a hill (https://mountainsensing.org/deployment/router-nodes/). In our case, our link did not arrive to one kilometer, mainly due to the obstacle the ground poses at that distance (the Fresnel radius to that distance is 9.29 m and the nodes are at 1.8 m above the ground).

Now, considering the maximum amount of packets that can be transmitted under the duty cycle regulation with short packets. Figure 8c shows that OFDM2-100 is the radio setting that can send up to 285 short packets within an hour. Not far behind we have 4FSK-FEC-200 and 2FSK-100 capable of transmitting 239 and 253 short packets per hour. 2FSK-50 can transmit only 138 short packets in the same period.

At this distance from TX, a few long packets can be transmitted per hour. The reference 2FSK-50 can transmit roughly 6 packets per hour while OFDM2-100 and OQPSK-50 just 10. We highlight those two PHYs. Therefore, if the user is interested in maximizing the throughput, 4FSK-FEC-200, 2FSK-100 and OFDM2-100 are the most performing PHYs to do so with short packet. With long packets, OFDM2-100 and OQPSK-50.

Figure 8e shows the average electric charge consumption per packet of 127 B transmitted. For short packets, the reference consumes 2178 μC whereas the less power-hungry in this case, OFDM2-100, consumes 964 μC.

For long packets, average power consumption is depicted in Figure 8f. 2FSK-50 consumes in average 47.5 mC. The less electric charge-consuming PHYs in this scenario are OFDM2-100 and OQPSK-50, with 26.8 mC and 29.1 mC respectively. Should the potential user be focused on reducing electric charge consumption, dotted-pattern highlighted bars in Figure 8e,f depict the less electric charge-hungry PHYs in this case.

### 4.2. Smart Agriculture Scenario

In this scenario, high data rates with high PDR are achieved up to 337 m, and maximum length of the radio link is reached around 615 m away from the TX node.

#### 4.2.1. RX at 337 m

**Figure 9:**
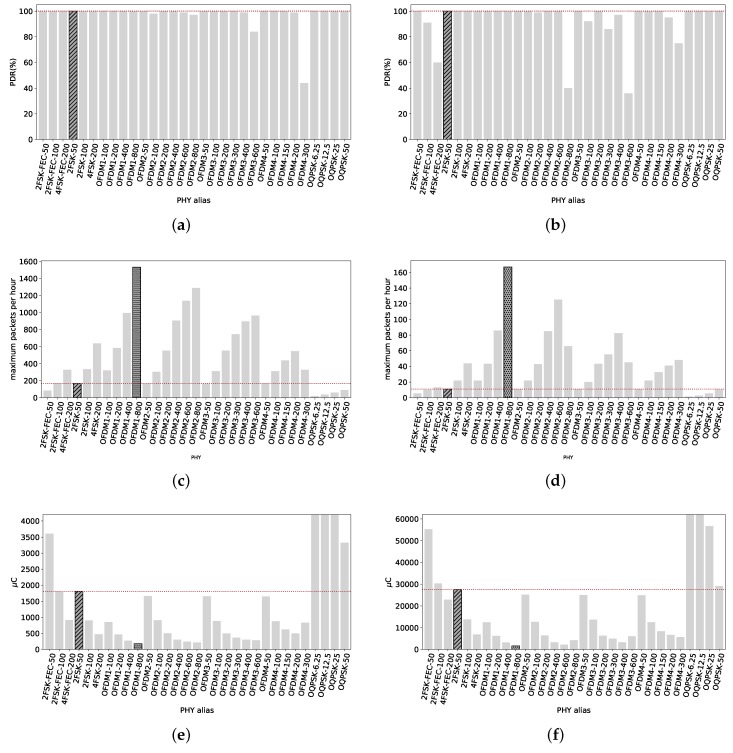
Smart Agriculture, 337 m. The PDR for all radio setting considering short and long packets is high. Using high data rates does not impact in the reliability while reducing electric charge consumption and increasing the number of packets exchange within the 0.1%duty cycle regulation. (**a**) Packets of 127 B. Almost all radio settings with a PDR close to 100%, including those with the higher data rates; (**b**) Packets of 2047 B. PDR of some radio settings drops, such as OFDM2-800, 2FSK-100 and 4FSK-200. The whole OFMD option 1 PHYs stay at 100%, as well as the reference 2FSK-50 and all the O-QPSK radio settings; (**c**) Maximum amount of short packets that can be transmitted per hour. OFDM1-800 can transmit up to 1531 packets while the reference just 166.; (**d**) Maximum amount of long packets that can be transmitted per hour. OFDM1-800 can transmit up to 167 packets, more than 10 times the reference with just 11 packets; (**e**) Average electric charge consumed per 127 B packet transmitted. OFDM1-800 is the less power-hungry, with 178 μC consumed, against the 1800 μC needed by 2SFK-50 to transmit the same packet. The rest of the high data rates OFDM PHYs also consume significantly less electric charge than the reference; (**f**) Average electric charge consumed to send a long packet. Still OFDM1-800 is the less electric charge-consuming, with 1637 μC per packet, whereas the reference consumes 27.5 mC.

With a packet size of 127 B, Figure 9a shows the PDR of almost all radio settings are very close to 100%, including those with the highest data rate possible (800 kbps). Increasing the packet size to 2047 B, Figure 9b shows that the PDR remains 100% for the majority of the radio settings, including OFDM1-800. 2FSK-50 is 100% in both, with short and long packets, as well as its variations with higher data rates. Therefore we do not highlight any other PHY, since the potential user has many options to choose a radio setting with 100% PDR.

We can see that the PDR values for some radio settings are higher for long packets than short packets. This is the case for OFDM2-200 and OFDM4-300 at 213 m, and for OFDM4-300 at 337 m. This can be linked to interference. Since we are transmitting packets with only 20 ms separation between them, short packets series are more likely to be affected than longer packets by a single transmission of a different radio technology device using the same frequency and with a lower data rate (a LoRa packet using Spreading Factor 12 with a 13 B payload has a ToA of 925 ms (LoRa Modem Calculator Tool)).

Figure 9c shows the maximum amount of packets that can be transmitted per hour, and we can see that OFDM1-800 can transmit up to 1531 packets while the reference 2FSK-50 only 166. We highlight OFDM1-800 since it can transmit at least 25% more packets than any other radio setting, and more that 9 times the amount of the reference. Considering long packets, OFDM1-800 has the capability of sending the maximum amount of packets per hour, up to 167 packets, while the reference roughly 11. Figure 9d shows the maximum amount of 2047 B packets that can be delivered at 337 m. In consequence, if the potential user wants to maximize the throughput of the nodes separated by a similar distance, OFDM1-800 offers the highest throughput.

Figure 9e depicts the average electric charge consumption per packet the radio needs in order to send a 127 B packet. The reference 2FSK-50 needs on average 1800 μC. The lowest amount of electric charge needed corresponds to OFDM1-800, with just 178 μC, around ten times less electric charge. Other radio settings are also “low energy” such as OFDM2-800 with 215 μC, and OFDM2-600 with 243 μC.

In Figure 9f, we can see the average electric charge consumed by the TX radio in order to transmit a long packet. The radio setting with the smallest electric charge requirement is OFDM1-800, with 1630 μC on average per a 2047 B transmitted packet. The reference 2FSK-50 consumes 27.5 mA, 16 times more than OFDM1-800. OFDM2-600 consumes 2200 μC, more than 12 times less than the reference. If the potential user wants to increase the lifetime of a battery power node, OFDM1-800 is the less electric charge demanding PHY in these conditions.

#### 4.2.2. RX at 615 m

At this distance, the overall performance of the radio settings is poor.

**Figure 10:**
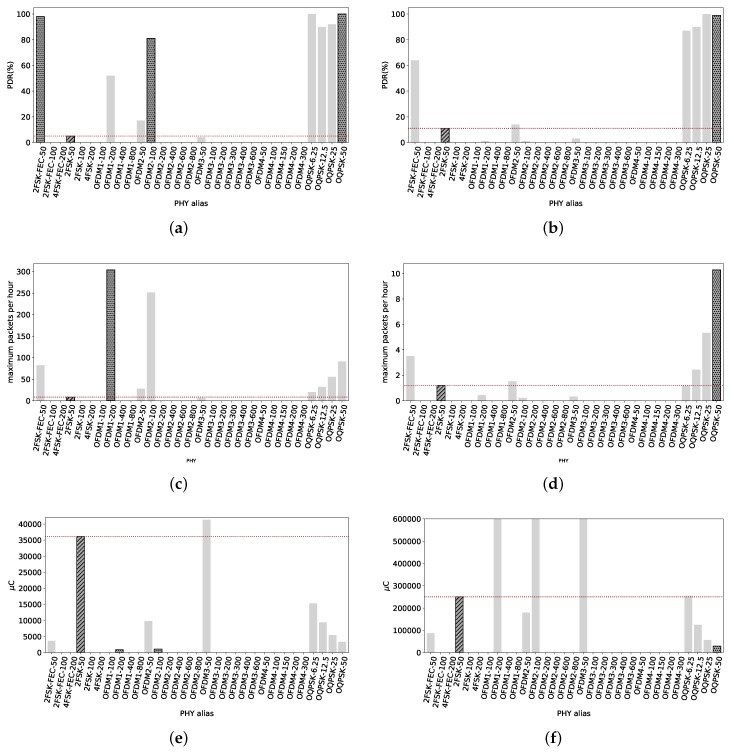
Smart Agriculture, 615 m. The radio link is very close to the maximum distance where it can deliver connectivity. High data rates OFDM radio settings are unable to get any packet across. Maximum data rate that can provide connectivity is 200 kbps, but with PDR of 52% with short packets. (**a**) PDR values for all radio settings with short packets. Only 2FSK-FEC-50, OFDM2-100 and all the O-QPSK radio settings have a PDR over 80%. The reference has a poor PDR, only 5%; (**b**) PDR values considering long packets. Only O-QPSK PHYs have PDR values over 85%, against the reference with 11%; (**c**) Maximum amount of TXs with short packets under 0.1% duty cycle. Despite its PDR of 52%, OFDM1-200 is able to transmit up to 300 short packets. The reference can send just 8 packets; (**d**) Maximum amount of TXs with long packets within one hour. OQPSK-50 can send up to 10 long packets while the reference roughly 1 packet per hour; (**e**) Energy consumed per 127 B packet transmitted. OFDM1-200 and OFDM2-100 are the most electric charge-efficient, consuming 895 μC and 1095 μC respectively. The reference consumes 36 mC. This is due to its poor PDR; (**f**) Energy consumed per 127 B packet transmitted. O-QPSK is the less electric charge-consuming radio setting, with 29.5 mC while the reference consumes 250 mC.

Figure 10a shows the PDR of all radio settings for short frames, where the majority of OFDM and FSK radio settings are not able to get any frames across. Only O-QSPK PHYs, 2-FSK-FEC-50 and OFDM2-100 have a PDR over 80%. The reference 2FSK-50 has a negligible PDR, only 5%.

PDR values for long packets are shown in Figure 10b. Only OQPSK PHYs maintain a high PDR. 2FSK-FEC-50 drops to 64% and the reference is only 11%. Therefore, if the potential user wants to have radio links with similar length, 2FSK-FEC-50, OFDM2-100 and any O-QPSK radio setting are useful for such requirement with short packets. For long packets, any O-QPSK radio setting is useful.

It can be seen in Table 8 that the PDR values at 538 m are lower than at 615 m. This seems counterintuitive since at further distance, lower should be the power of the received signal and thus, the PDR. This assumption is correct in environments where the attenuation of the radio signals are only due to distance, without obstacles located between the transmitter and receiver. But this is not our case. The obstacles surrounding the RX node located at 538 m and its relative position to the TX node cause this shadowing effect, affecting its PDR. At 615 m, the surroundings of the RX node did not have this effect and therefore, the PDR values are higher.

The maximum amount of short packets that can be sent under the 0.1% duty cycle regulation are shown in Figure 10c. Even though OFDM1-200 has only 52% PDR, its high data rate allows it to send just over 300 packets per hour. Not far behind, OFDM2-100 has the possibility of sending up to 251 packets per hour. 2FSK-50 only 8 packets, and OQPSK-50 below 100 packets of 127 B.

Now considering long packets, Figure 10d shows the maximum amount each PHY can correctly send within an hour. 2FSK-50 can send one long packet per hour, whereas OQPSK-50 just 10. For a potential user, if it is wanted to maximize the amount of transmitted packets, OFDM1-200 is the best option for short packets. For long packets, OQPSK-50 is the best option but just a few packets possible.

Figure 10e shows the electric charge consumed on average per short packet transmitted to this node. 2FSK-50 has a poor PDR, thus the eventual need for retransmissions makes energetically expensive to get a packet across. It consumes 36 mC, whereas OFDM1-200 and OFDM2-100 consume 895 μC and 1095 μC respectively. Therefore, we highlight these last two as the more efficient in electric charge consumption. For long packets, Figure 10f shows the average consumption. The reference 2FSK-50 consumes 250 mC per packet of 2047 B. We can observe that the less consuming PHY is OQPSK-50, with 29.5 mC. In both cases, for short and long packets, 2FSK-50 is at least one magnitude higher than the most electric charge-efficient PHYs.

### 4.3. Urban Canyon Scenario

High data rates with high PDR values are reached up to 540 m away from the TX node. The maximum length of the radio link is at 779 m, with important losses at 602 m and 742 m due to obstacles and the topography of the scenario.

#### 4.3.1. RX at 540 m

**Figure 11:**
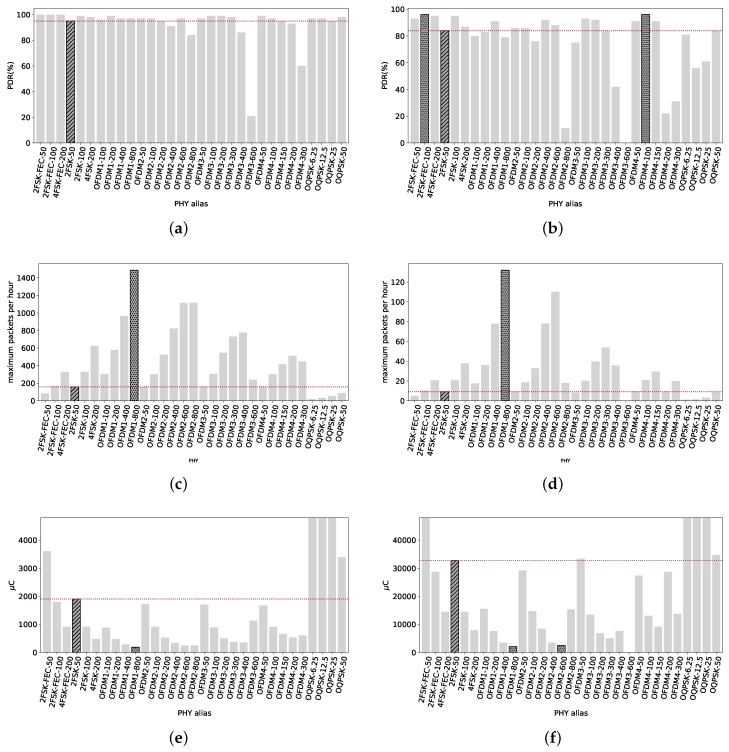
Urban Canyon, 540 m. Some high data rate radio settings have a good PDR, close to 100%, and some others suffer from interferences. This is expected in this type of scenarios so an agile MAC layer would be useful in order to overcome those interferences by either changing frequency or waiting for the availability of the medium. (**a**) PDR values with packets of 127 B. Overall, high reliability except for OFDM3-600 and OFDM4-300. Reference value, 98% PDR; (**b**) PDR values with 2047 B packets. Overall values are over 50%, with a few OFDM radio settings. Highest values with 2FSK-FEC-100 and OFDM4-100, 95% PDR and 96% PDR. Reference value, 84% PDR; (**c**) Maximum amount of TX packets of 127 B under the 0.1% duty cycle regulation. OFDM1-800 can transmit up to 1485 packets, while the reference 158 packets. OFDM2-600 and OFDM2-800 can also transmit more than 1000 short packets; (**d**) Maximum amount of TX packets of 2047 B under the 0.1% duty cycle regulation. OFDM1-800 can send up to 132 packets, OFDM2-600 110 packets and the reference just 9 packets; (**e**) Average electric charge consumed per packet of 127 B sent. OFDM1-800 is the less consuming radio setting, with 184 μC per packet whilst 2FSK-50 consumes 1900 μC; (**f**) Average electric charge consumed per packet of 2047 B sent. OFDM1-800 and OFDM2-600 are the more electric charge-efficient PHYs, consuming around 2.1 mC and 2.5 mC. 2FSK-50 consumes around 33 mC.

PDR values of all radio settings are depicted in Figure 11a for short packets. Overall, all PHYs have a high reliability, with values between 84% and 100% PDR. The exceptions are OFDM3-600 and OFDM4-300, with values of 21% and 60% PDR respectively. The reference 2FSK-50 has 95% PDR. We do not highlight any since there are many PHYs with their PDR greater that the reference.

For long packets, the PDR values are shown in Figure 11b. We can see the PDR values decay a few points, but still several PHYs with higher PDR that the reference (with 84% PDR). The two PHY with highest reliability with long packets are 2FSK-FEC-100 and OFDM4-100 with 95% and 96% PDR respectively. Noticeably, O-QPSK radio settings have a lower PDR, even though their sensitivity is higher. Their low data rate makes the time needed to send each frame longer, increasing the probabilities of collision with other networks/technologies during transmissions.

Figure 11c shows the maximum number of packets that can be sent under the 0.1% duty cycle regulation. OFDM1-800 stands alone with the possibility of sending 1485 packets. Some hundreds of packets behind, OFDM2-600 and OFDM2-800 with roughly 1115 packets. The reference 2FSK-50 can send up to 158 packets per hour.

Figure 11d shows the maximum number of long packets that can be sent per hour. Still, OFDM1-800 is the PHY that allows the maximum number of transmissions per hour, under the duty cycle regulation, with roughly 132 packets. OFDM2-600 allows 110 full packet length transmissions, while the reference only 9. Consequently, if the potential user wants to maximize the amount of packets until the duty cycle regulation limit, OFDM1-800 is shown to be the best for short and long packets.

The average amount of electric charge used to transmit a 127 B packet for all the radio settings can be seen in Figure 11e. The lower electric charge consumption to get one packet across is achieved with OFDM1-800, spending 184 μC. The reference 2FSK-50 spends 1900 μC, one magnitude higher.

Concerning the electric charge consumption for long packets, Figure 11f shows these values. The tendency is maintained, OFDM1-800 is the most efficient radio setting to send a packet of 2047 B, with an average consumption of 2070 μC. OFDM2-600 is close to this value, with 2507 μC per 2047 B sent. 2FSK-50 consumes, for the same transmission, around 32,760 μC.

#### 4.3.2. RX at 779 m

**Figure 12:**
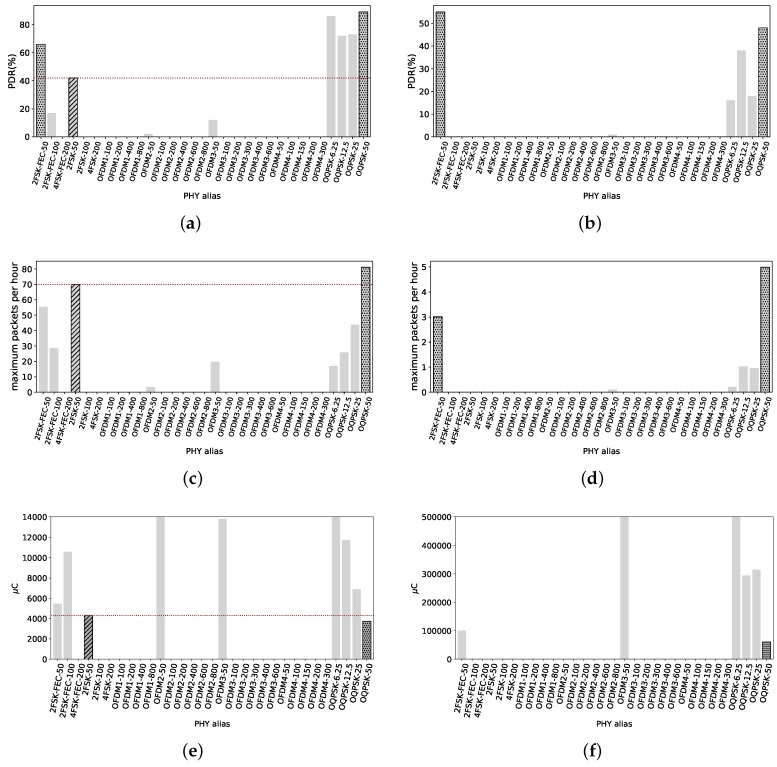
Urban Canyon, 779 m. The limit of the radio link is very close to this distance of 779 m from the TX node. The PDR for short and long packets is poor, so is the maximum amount of packets per hour. Applications with short packets and low throughput can stand radio links with similar characteristics. (**a**) PDR with packets of 127 B. Only data rates up to 50 kbps provide some connectivity, with the exception of O-QPSK-50 whose PDR is above 80%. We are very close to the maximum length of the radio link. PDR of 2FSK-50 is 42%; (**b**) PDR with packets of 2047 B. Only 2FSK-FEC-50 has a PDR over 50%. The rest of the radio settings does not provide any connectivity, with the exception of the O-QPSK radio settings; (**c**) Maximum amount of short packets transmitted per hour. The reference 2FSK-50 can send up to 70 packets and OQPSK-50 81 packets; (**d**) Maximum amount of long packets transmitted per hour. OQPSK-50 can send up to 5 2047 B packets per hour under the 0.1% duty cycle regulation; (**e**) Average electric charge consumption per 127 B packet sent. 2FSK-50 needs 4.3 mC to transmit one short packet whilst OQPSK-50 consumes 3.7 mC per packet; (**f**) Average electric charge consumption per packet of 2047 B sent. O-QSPK consumes 60.7 mC, less than any other radio setting.

At this distance we approach to the limit of the radio link. As shown in Figure 12a, only radio settings with data rates ≤ 50 kbps present PDR values over 60% for frames of 127 B. The PDR of the reference 2FSK-50 is only 42%. 2FSK-FEC-50 is the only non O-QPSK PHY with its PDR > 50%. This RX node is located at the limits of the coverage proportionated by the TX node. We can observe the PDR values in Figure 12b for packets of 2047 B. Only 2FSK-FEC-50 has a PDR over 50%. Even OQPSK PHYs, with their higher sensitivity, do not have a PDR over 50%. At this distance and in these conditions, only short packets can deliver a good reliability of 80% with OQPSK-50.

Figure 12c shows the maximum amount of 1287 B packets that can be sent within an hour under the 0.1% duty cycle regulation. Only OQPSK-50 outperforms the reference, with the possibility of sending up to 81 packets per hour against 70 packets of 2FSK-50. For long packets, the maximum amount of packets per hour can be seen in Figure 12d. With a maximum of roughly 5 packets per hour, OQPSK-50 is the most convenient PHY. 2FSK-FEC-50 can send up to just 3 full packets and 2FSK-50 is incapable of get any packet across.

Figure 12e shows the average electric charge consumed by the TX node in order to get a 127 B packet across with the different PHYs. 2FSK-50 consumes 4.3 mC and OQPSK-50 consumes 3.7 mC. Any other PHY does not consume less electric charge than those two.

Similarly but with long packets, we see the electric charge consumption in Figure 12f. The TX node needs 105 mC to get a packet across when using 2FSK-FEC-50 and 60.7 mC with OQPSK-50. In this scenario and at this distance, low data rates and poor PDR make energetically expensive to get packets correctly transmitted. If compared with the values at 540 m, at 779 m it is consumed twice the electric charge (2FSK-50 at 540 m consumes 32 mC vs. OQPSK-50 at 779 m with 60.7 mC).

In this scenario, we can see results that are counterintuitive:

For OFDM1-800 and OFDM2 PHYs at 406 m, the PDR is lower for low data rates (50 to 400 kbps) than for high data rates (600 and 800 kbps). In interference-and-obstacle-free scenarios, it should be the opposite since lower data rates PHYs have a higher sensitivity. This can be attributed to interference occurred at the moment of the experiment. This affirmation is backed by the fact that for the rest of the radio settings at this distance, the PDR values are above 90%, even for those PHYs with lower sensitivity. Same reasoning goes with OFDM2-400 and OFDM2-600 at 512 m.

Short frames have worst PDR than long frames. The reason is also interference, since a burst of short packets can be more affected by a single transmission of different technology/network using a lower data rated communication than a long packet burst. We see this more notably in OFDM2-100 at 406 m and OFDM1-400 at 512 m.

Nodes located at further distance presenting higher PDR values than nodes located closer to the TX node. We are in a scenario where the radio signals interact with trees, people, cars, buses, shops, buildings and big metal infrastructure and for some RX locations, these objects are in the way between the TX and RX nodes. These objects can cause the shadowing effect of the node, affecting the quality of the link between TX and RX and thus, drastically reducing the PDR for that link.

### 4.4. Advanced Metering Infrastructure Scenario

For the nodes located within 215 m, the PDR values of almost all radio settings are close to 100%. Taking one of those 4 locations would have similar results to those exposed in Section 4.3.1. For the RX nodes at 350 m and 400 m, PDR values decay and we approach to the limits of the radio link. Therefore we present the analysis for those 2 RX locations. Several buildings are between TX and those 2 RX nodes, making it a challenging environment to the radio link.

#### 4.4.1. RX at 350 m

**Figure 13:**
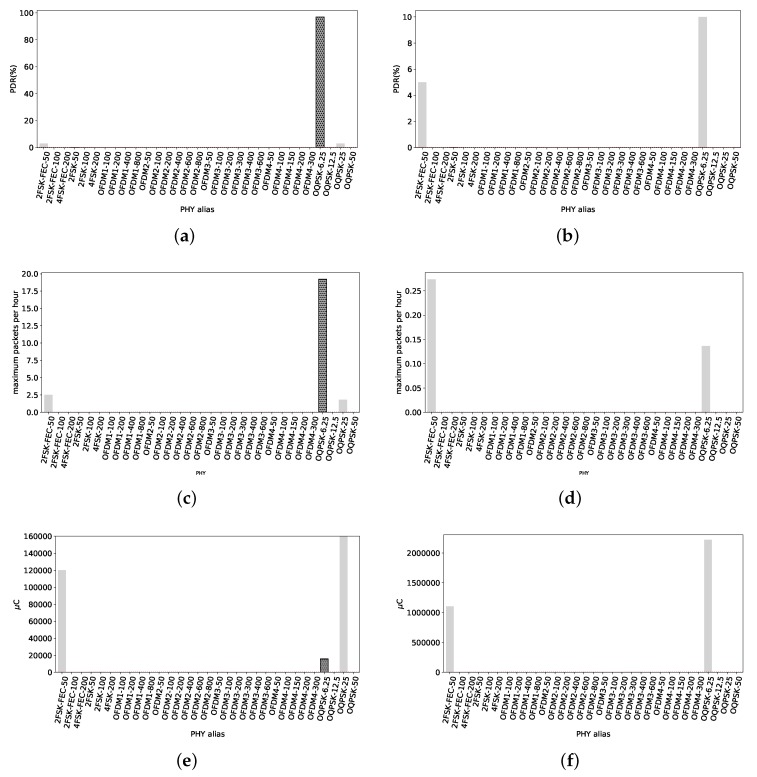
AMI, 350 m. Only the radio setting OQPSK-6.25 with short packets is useful for this type of challenging conditions, with several buildings between nodes. (**a**) PDR values considering packets of 127 B. Only OQPSK-6.25 can provide good connectivity with 97% PDR. The rest of the radio links offers no connectivity at all, PDR values of 0%; (**b**) PDR values with long packets of 2047 B. The quality of the radio link is overall poor. With PDR values below 10%, no communication link is possible; (**c**) Maximum amount of TX packets per hour. OQPSK-50 can roughly send 19 packets per hour. 2FSK-FEC-50 only 2 and OQPSK-25 only 1. The rest of the PHYs cannot get even one packet across; (**d**) Maximum amount of TX packets per hour. Any radio setting is able to send one packet; (**e**) Average electric charge consumed per packet of 127 B transmitted. OQPSK-6.25 needs 15.7 mC per short packet; (**f**) Average electric charge consumed per packet of 2047 B transmitted. On average, it takes more than 1 C per packet using 2FSK-FEC-50 and more than 2 C if OQPSK-6.25 is used.

Figure 13a shows the PDR values for all radio settings with a packet length of 127 B. The radio link is almost nonexistent. Only OQPSK-6.25, the PHY with the slowest data rate available in the standard, has a high PDR value, 97%. The rest of the radio settings have a negligible PDR. Figure 13b depicts the poor PDR values with long packets. It is not possible to establish a radio communication with these PDR values, which is as high as 10% for OQPSK-6.25.

Figure 13c shows the maximum number of packets that can be sent within an hour. OQPSK-6.25 is capable of delivering roughly 19 short packets. This is enough for some applications where few sensor readings are performed per hour. Considering long packets, it is not possible to get even one packet across for any PHY, as it is shown in Figure 13d.

The average electric charge consumed to transmit a packet is shown in Figure 13e. With OQPSK-6.25, the TX node consumes 15.7 mC per packet of 127 B to get correctly sent. For long frames, it would take more than 2 C to get a packet across. Therefore, long frames in this scenario are not achievable.

#### 4.4.2. RX at 400 m

**Figure 14:**
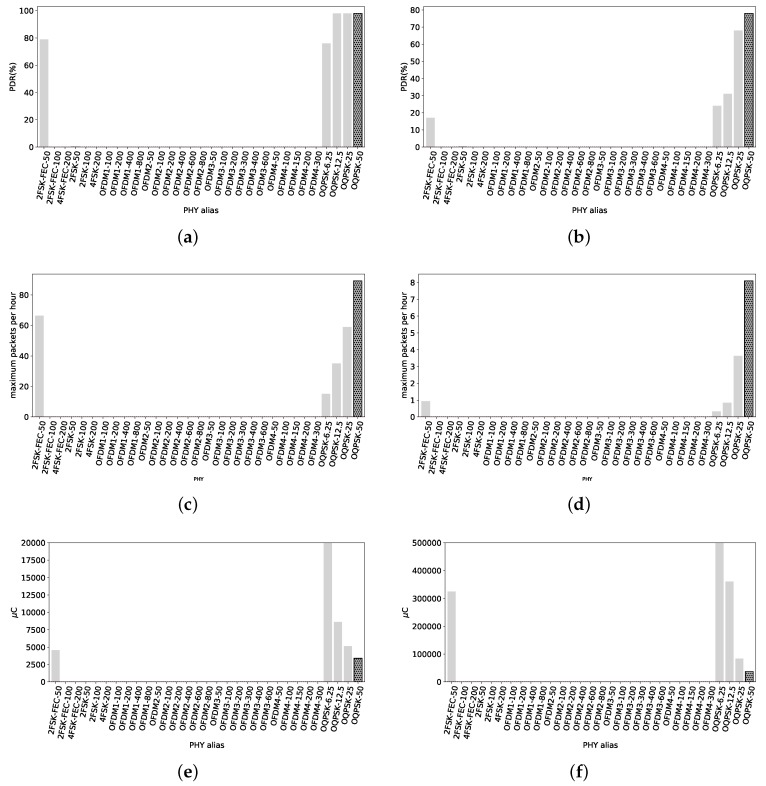
AMI, 400 m. It is normal to have several buildings and any other kind of obstacles between nodes. Having these conditions, the most useful radio setting is OQPSK-50. (**a**) PDR values with packets of 127 B. OQPSK-12.5, OQPSK-25 and OQPSK-50 have 98% PDR. We see that the most advantageous of that group is OQPSK-50, offering same reliability with higher data rate; (**b**) PDR values with packets of 2047 B. OQPSK-50 offers the highest reliability, 78%, between the 5 radio settings with non-zero PDR; (**c**) Maximum amount of TX short packets under 0.1% duty cycle regulation. O-QPSK-50 can send up to 90 short packets, more than any other radio setting; (**d**) Maximum amount of TX long packets under 0.1% duty cycle regulation. OQPSK-50 is able to send up to 8 long packets within one hour; (**e**) Average electric charge consumed per packet of 127 B transmitted. OQPSK-50 consumes on average, 3.4 mC per packet transmitted; (**f**) Average electric charge consumed per packet of 2047 B transmitted. OQPSK-50 is the less electric charge-hungry radio setting, consuming 37 mC per packet transmitted.

Figure 14a depicts the PDR values for short packets. 2FSK-FEC-50 and O-QPSK PHYs present PDR values above 75%. The rest of the PHYs do not have any reliability, PDR values are zero. OQPSK-12.5, OQPSK-25 and OQPSK-50 have 98% PDR. In Figure 14b is shown the PDR for 2047 B packets. The PDR of 2FSK-FEC-50 decays below 20% while the PDR of OQPSK-50 is still high, at 78%. Therefore, the radio setting with the highest reliability for short and long packets is OQPSK-50 and we highlight it.

Figure 14c shows the maximum number of short packets that can be transmitted. The radio setting that can transmit more packet within one hour is OQPSK-50 with roughly 90 packets. Now considering long packets, see Figure 14d, OQPSK-50 is still the PHY with a maximum of 8 possible packets per hour. Ergo, in order to maximize the throughput of the nodes and being compliant with the duty cycle regulation, OQPSK-50 is the most convenient.

It is depicted in Figure 14e the average electric charge consumed per short packet transmitted. The less power-hungry is the OQPSK-50 PHY, with 3.4 mC. Similarly, Figure 14f shows the electric charge consumption for long packets. The tendency is maintained, OQPSK-50 consumes the less amount of electric charge per 2047 B packet, with 37 mC. Thus, OQPSK-50 is the more electric charge-efficient PHY for this scenario, despite of the length of the packet.

## 5. Discussion

The lower the frequency, the higher the maximum coverage of the signal with the same TX power (considering devices with similar sensitivity). Consequently, the use of a frequency band below 2.4 GHz increases the range of the radio signals. In comparison with a 2.4 GHz solution, sub-GHz enables networks with fewer hops, allowing simpler deployments but with the performance and flexibility that mesh networks provide.

The adoption of the PHYs described in the “g” amendment of IEEE802.15.4 can be beneficial to any type of WSN due to the following reasons:

- They can be used in sub-GHz bands, increasing the range of the radio links.
- Higher data rates, up to 800 kbps, reduce the transmission/reception time for a packet; this lowers the electric charge consumption per byte exchanged and increases the maximum amount of packets that can be exchanged considering duty cycle regulations.
- They provide long range as a Low-Power Wide Area Network (LPWAN) combined with the flexibility of a mesh network. This enables networks to be built with simple architectures (few-hop deep networks) bringing good trade-off between performance and simplicity.
- The diverse characteristics of each modulation enable further optimization to the low-power networks solutions. Networks are now able to trade-off data rate, robustness, electric charge consumption, range and duty cycle. Choices on the PHY to be used can be made according to the current conditions.

Regulations on sub-GHz bands are in constant revision, which poses an additional challenge to keep up with all the evolutions.

This work is meant to provide the reader a first reference of what can be achieved by the IEEE802.15.4g standard in terms of PDR and range on different scenarios where WSN are likely to be deployed. We acknowledge that the ideal approach would be to run the experiments several times, well separated in time in order to provide results richer in statistical relevance. By doing this, we can reduce the impact that transient effects may have in the results. In addition, experiments should be repeated with the RX nodes located half wavelength away in several directions from the selected measurement point. This would be useful in order to discard positions where multi-path fading can heavily attenuate the radio signal.

Nonetheless, these empirical results are taken in real-world scenarios. This provides a snapshot of the performance of IEEE802.15.4g at the precise moment of the experiment, including few inconsistencies in some measurements due to external factors, as a real deployment would encounter. To the best of our knowledge, these are the first experiments exploring the entire IEEE802.15.4g standard.

### 5.1. On the Longer Range of FSK-FEC and O-QPSK

Throughout the experiments we can observe that the longest radio links were obtained when using the FSK-FEC and O-QPSK PHYs. This is expected as both set of PHYs have the highest sensitivity of all PHYs, delivering up to 141 dB of link budget (see Table 6, OQPSK-6.25).

OFDM radio signals are more robust against interferences and multi-path effects. OFDM1-100 uses 104 sub-carriers (96 data and 8 pilot tones) with 4x frequency repetition (there are 4 sub-carriers with the same information), providing a high level of robustness. OFDM2-50 has the same characteristics but half of the sub-carriers and thus, half of the data rate.

Each OFDM option 1 channel occupies 1.2 MHz and the 4 equal sub-carriers are separated by the same distance (in frequency). Even if 3 sub-carriers get compromised during one symbol transmission on the path towards the receiver, this can still recover the information. Even though that robustness is provided, in Table 7 the PDR for this PHY is just 31% and 5% for short and long packets at 700 m. For OFDM2-100, the PDR increased to 92% and 47%. This PHY is 2 dB more sensitive than OFDM1-100. We see in Table 6 that the maximum power for OFDM is +11 dBm whereas for the rest is +14 dBm.

Our believe is that the longer range of the O-QPSK and FSK-FEC PHYs can be attributed to their higher sensitivity in comparison to the rest of the PHYs tested. FSK-FEC holds 5 dB, 5 dB and 8 dB higher sensitivity than its non-FEC counterpart (2FSK-50, 2FSK-100, 4FSK-200). When compared 2FSK-50 with OQPSK-50, the difference is 8 dB.

The reason why OFDM PHYs do not reach as far as these PHYs is that they present a lower sensitivity and a lower maximum TX power.

These values of sensitivity and maximum TX power are directly dependent of the hardware and can vary depending on the manufacturer.

### 5.2. Future Work

Having such diverse options of PHYs available, it is possible to build a mesh network with heterogeneous radio settings. We see the need to design an agile MAC layer with the following characteristics:

- capable of choosing the most convenient PHY for each pair of nodes.
- with a Time-Slotted Channel Hopping (TSCH) approach, to reduce power consumption by eliminating idle listening and reduce collision probabilities. This adds a level of determinism, therefore nodes switch on their radios only when their schedule indicate that they are expected to transmit/receive frames. The rest of the time nodes can sleep, saving electric charge.
- a CCA procedure, to be able to increase the duty cycle from 0.1% to the maximum 2.8%.

## Figures and Tables

**Table 1:** Radio settings using FSK modulation. The values of these parameters are defined in the standard [22]. Data rate values consider the amount of bits per second leaving the radio. In the case where FEC is used, 1 bit of information is encoded in 2 bits leaving the radio, therefore the information rate reduces to half. Channel spacing is the separation between the center of two adjacent channels. The “Alias” column provides an identifier to each group of parameters.

PHY	FEC 1/2 Rate	Data Rate	Ch. Spacing	Alias
2-FSK	No	50 kbps	200 kHz	2FSK-50
2-FSK	No	100 kbps	400 kHz	2FSK-100
4-FSK	No	200 kbps	400 kHz	4FSK-200
2-FSK	Yes	50 kbps	200 kHz	2FSK-FEC-50
2-FSK	Yes	100 kbps	400 kHz	2FSK-FEC-100
4-FSK	Yes	200 kbps	400 kHz	4FSK-FEC-200

**Table 2:** Radio settings using OFDM modulation. The values of these parameters are defined in the standard [22]. Channel spacing is the separation between the centers of two adjacent channels. OFDM channels group a number of sub-carriers, option 1 being the widest one (the most number of sub-carriers), option 4 the narrowest (the least number of sub-carriers). The data rate depends on the combination of the MCS (Modulation and Coding Scheme) value and the OFDM option. Frequency repetition identifies the number of sub-carriers encoding the same information. The “Alias” of each radio setting is used to identify each set of parameter, e.g., OFDM1-400 identifies the PHY using OFDM option 1 and MCS2.

Parameter	OFDM Opt. 1	OFDM Opt. 2	OFDM Opt. 3	OFDM Opt. 4
Channel spacing (kHz)	1200 kHz	800 kHz	400 kHz	200 kHz
Number of sub-carriers	104	52	26	14
MCS0 (kbps) - 4x freq. rep.	100 kbps	50 kbps	-	-
MCS1 (kbps) - 2x freq. rep.	200 kbps	100 kbps	50 kbps	-
MCS2 (kbps) - 2x freq. rep.	400 kbps	200 kbps	100 kbps	50 kbps
MCS3 (kbps) - 2x freq. rep.	800 kbps	400 kbps	200 kbps	100 kbps
MCS4 (kbps) - No freq. rep.	-	600 kbps	300 kbps	150 kbps
MCS5 (kbps) - No freq. rep.	-	800 kbps	400 kbps	200 kbps
MCS6 (kbps) - No freq. rep.	-	-	600 kbps	300 kbps
Alias	OFDM1-<datarate>	OFDM2-<datarate>	OFDM3-<datarate>	OFDM4-<datarate>

**Table 3:** Radio settings using O-QPSK modulation. The values of these parameters are defined in the standard [22]. The rate mode defines the resulting data rate. The radio signal is composed by chips; in the 868 MHz frequency band, the standard uses a fixed 100 kchip/s. The spreading mode defines the amount of chips needed to encode a bit of information.

Rate Mode	Chip Rate kchip/s	Data Rate kbps	Alias
0	100	6.25 kbps	OQPSK-6.25
1	100	12.5 kbps	OQPSK-12.5
2	100	25 kbps	OQPSK-25
3	100	50 kbps	OQPSK-50

**Table 4:** EU Harmonized NRI for the 863–870 MHz band. A device following these rules can be used across Europe. This band is divided into sub-bands, each with a specific regulation. Channel access and occupation rules refers to the duty cycle that devices must respect. Each sub-band also defines the maximum bandwidth a channel can have. The other usage restrictions column indicates the applications that can take place and the transmission techniques than can be used. DSSS is short for Direct Sequence Spread Spectrum, FHSS is short Frequency Hopping Spread Spectrum.

Frequency Band	Maximum Effective Radiated Power (e.r.p.)	Channel Access and Occupation Rules	Maximum Occupied Bandwidth	Other Usage Restrictions
863–865 MHz	25 mW e.r.p.	≤0.1% duty cycle or polite spectrum access	the entire band except audio/video apps. limited to 300 kHz	
865–868 MHz	25 mW e.r.p. +6.2 dBm/100 kHz	≤1% duty cycle or polite spectrum access	the entire band except audio/video apps. limited to 300 kHz	DHSS and any techniques other than FHSS
868.0–868.6 MHz	25 mW e.r.p.	≤1% duty cycle or polite spectrum access	the entire band except audio/video apps. limited to 300 kHz	
868.7–869.2 MHz	25 mW e.r.p.	≤0.1% duty cycle or polite spectrum access	the entire band except audio/video apps. limited to 300 kHz	
869.40–869.65 MHz	25 mW e.r.p.	≤0.1% duty cycle or polite spectrum access	the entire band	Analogue audio apps. other than voice excluded. Analogue video apps excluded.
869.40–869.65 MHz	500 mW e.r.p.	≤0.1% duty cycle or polite spectrum access	the entire band	Analogue video apps. are excluded.
869.7–870 MHz	5 mW e.r.p.	No requirement	the entire band	Audio and video apps. are excluded.
869.7–870 MHz	25 mW e.r.p.	≤1% duty cycle or polite spectrum access	the entire band	Analogue audio apps. are excluded. Analogue video apps. are excluded.

**Table 5:** Non-EU-wide harmonized National Radio Interfaces. Some European countries accept the use of the 863–870 MHz band with these characteristics. Channel access and occupation rules refers to the duty cycle that devices must respect. The other usage restrictions column indicates the applications that can take place and the transmission techniques than can be used.

Frequency Band	Maximum Effective Radiated Power (e.r.p)	Channel Access and Occupation Rules	Maximum Occupied Bandwidth	Other Usage Restrictions
863–870 MHz	25 mW e.r.p	≤ 0.1% duty cycle or polite spectrum access	the entire band except audio/video limited to 300 kHz and voice to 25 kHz	sub-bands 868.6–868.7 MHz, 869.25–869.4 MHz and 869.65–869.7 MHz can only be used for alarm systems

**Table 6:** Radio characteristics of the ATREB215 XPRO Extension board featuring the AT86RF215 radio chip. For each item in the PHY Alias column (indicating a radio setting), the table specifies the maximum TX power allowed by the hardware, its current consumption when transmitting at maximum power, the receiver sensitivity with the condition in which this value is obtained and the link budget for each PHY (considering the 2 dBi antennas connected to the radios). PSDU stands for Packet Service Data Unit and is the PHY payload. PER stands for Packet Error Rate. TX power was set to the maximum allowed by the hardware.

PHY Alias	Max TX Power	Current Consumption @max TX power	Receiver Sensitivity	Sensitivity Condition	Link Budget
2FSK-50	+14 dBm	84.1 mA	−109 dBm	PSDU length	127 dB
2FSK-100	+14 dBm	83.9 mA	−106 dBm	250 B	124 dB
4FSK-200	+14 dBm	83.6 mA	−96 dBm	PER < 10%	114 dB
2FSK-FEC-50	+14 dBm	83.7 mA	−114 dBm		132 dB
2FSK-FEC-100	+14 dBm	83.6 mA	−111 dBm		129 dB
4FSK-FEC-200	+14 dBm	83.7 mA	−104 dBm		122 dB
OFDM1-100	+10 dBm	75.6 mA	−109 dBm	PSDU length	123 dB
OFDM1-200	+10 dBm	75.6 mA	−109 dBm	250 B	123 dB
OFDM1-400	+10 dBm	75.6 mA	−107 dBm	PER < 10%	121 dB
OFDM1-800	+10 dBm	76 mA	−104 dBm		118 dB
OFDM2-50	+10 dBm	76.5 mA	−111 dBm		125 dB
OFDM2-100	+10 dBm	76.5 mA	−111 dBm		125 dB
OFDM2-200	+10 dBm	76.7 mA	−108 dBm		122 dB
OFDM2-400	+10 dBm	76.7 mA	−106 dBm		120 dB
OFDM2-600	+10 dBm	76.8 mA	−104 dBm		125 dB
OFDM2-800	+10 dBm	77.1 mA	−101 dBm		115 dB
OFDM3-50	+10 dBm	76 mA	−113 dBm		127 dB
OFDM3-100	+10 dBm	76.1 mA	−109 dBm		123 dB
OFDM3-200	+10 dBm	76.1 mA	−107 dBm		121 dB
OFDM3-300	+10 dBm	75.3 mA	−106 dBm		120 dB
OFDM3-400	+10 dBm	75.8 mA	−102 dBm		116 dB
OFDM3-600	+10 dBm	76 mA	−97 dBm		111 dB
OFDM4-50	+11 dBm	75.8 mA	−111 dBm		126 dB
OFDM4-100	+11 dBm	75.8 mA	−109 dBm		124 dB
OFDM4-150	+11 dBm	75.8 mA	−108 dBm		123 dB
OFDM4-200	+11 dBm	75.8 mA	−105 dBm		120 dB
OFDM4-300	+11 dBm	75.8 mA	−101 dBm		116 dB
OQPSK-6.25	+14 dBm	84.1 mA	−123 dBm	PSDU length 20 B	141 dB
OQPSK-12.5	+14 dBm	84.1 mA	−121 dBm	PER < 10%	139 dB
OQPSK-25	+14 dBm	84.1 mA	−119 dBm		137 dB
OQPSK-50	+14 dBm	84.1 mA	−117 dBm	PSDU length 250 B	135 dB
				PER < 10%	

**Table 7:** Line of Sight. PDR considering packets of 127 B and 2047 B for each RX node. High data rates with high PDR are achieved at least up to 420 m from the TX node. Maximum length of the radio link is close to 700 m.

PHY Alias	RX at 420 m PDR 127 B–2047 B	RX at 700 m PDR 127 B–2047 B	RX at 1000 m PDR 127 B–2047 B
2FSK-50	100%–100%	83%–58%	0%–0%
2FSK-100	100%–100%	76%–0%	0%–0%
4FSK-200	100%–99%	0%–0%	0%–0%
2FSK-FEC-50	100%–100%	95%–94%	0%–0%
2FSK-FEC-100	100%–100%	100%–79%	0%–0%
4FSK-FEC-200	100%–100%	73%–38%	0%–0%
OFDM1-100	100%–100%	31%–5%	0%–0%
OFDM1-200	100%–100%	0%–18%	0%–0%
OFDM1-400	100%–100%	0%–0%	0%–0%
OFDM1-800	100%–100%	0%–0%	0%–0%
OFDM2-50	99%–98%	23%–59%	0%–0%
OFDM2-100	100%–98%	92%–47%	0%–0%
OFDM2-200	100%–100%	0%–0%	0%–0%
OFDM2-400	100%–99%	0%–0%	0%–0%
OFDM2-600	100%–100%	0%–0%	0%–0%
OFDM2-800	98%–51%	0%–0%	0%–0%
OFDM3-50	100%–100%	34%–26%	0%–0%
OFDM3-100	100%–100%	0%–0%	0%–0%
OFDM3-200	100%–100%	0%–0%	0%–0%
OFDM3-300	100%–100%	0%–0%	0%–0%
OFDM3-400	100%–97%	0%–0%	0%–0%
OFDM3-600	26%–0%	0%–0%	0%–0%
OFDM4-50	100%–98%	35%–11%	0%–0%
OFDM4-100	98%–99%	1%–1%	0%–0%
OFDM4-150	100%–99%	8%–0%	0%–0%
OFDM4-200	99%–100%	0%–0%	0%–0%
OFDM4-300	97%–63%	0%–0%	0%–0%
OQPSK-6.25	100%–99%	100%–98%	27%–1%
OQPSK-12.5	100%–100%	94%–94%	2%–1%
OQPSK-25	100%–100%	100%–83%	0%–0%
OQPSK-50	100%–99%	100%–100%	0%–0%

**Table 8:** Smart Agriculture. PDR for packets of 127 B and 2047 B for each RX node. Maximum coverage with high data rate happens at 337 m and maximum length of the radio link with useful PDR and data rate of at least 50 kbps is at 615 m.

PHY Alias	RX at 213 m PDR 127 B–2047 B	RX at 337 m PDR 127 B–2047 B	RX at 439 m PDR 127 B–2047 B	RX at 538 m PDR 127 B–2047 B	RX at 615 m PDR 127 B–2047 B	RX at 715 m PDR 127 B–2047 B
2FSK-50	100%–96%	100%–100%	31%–33%	0%–0%	5%– 11%	0%–0%
2FSK-100	100%–100%	100%–100%	0%–2%	0%–0%	0%–0%	0%–0%
4FSK-200	99%–100%	100%–100%	0%–0%	0%–0%	0%–0%	0%–0%
2FSK-FEC-50	100%–98%	100%–100%	100%–100%	0%–33%	98%–64%	0%–0%
2FSK-FEC-100	100%–100%	100%–91%	85%–32%	23%–6%	0%–0%	0%–0%
4FSK-FEC-200	100%–100%	100%–60%	0%–0%	0%–0%	0%–0%	0%–0%
OFDM1-100	100%–99%	100%–100%	0%–0%	0%–0%	0%–0%	0%–0%
OFDM1-200	100%–99%	100%–100%	0%–0%	0%–0%	52%–1%	0%–0%
OFDM1-400	100%–100%	100%–100%	0%–0%	0%–0%	0%–0%	0%–0%
OFDM1-800	100%–100%	100%–100%	0%–0%	0%–0%	0%–0%	0%–0%
OFDM2-50	100%–100%	100%–100%	5%–5%	0%–0%	17%–14%	0%–0%
OFDM2-100	100%–99%	98%–100%	0%–0%	0%–0%	81%–1%	0%–0%
OFDM2-200	73%–98%	100%–99%	0%–0%	0%–0%	0%–0%	0%–0%
OFDM2-400	99%–100%	100%–100%	0%–0%	0%–0%	0%–0%	0%–0%
OFDM2-600	99%–99%	99%–100%	0%–0%	0%–0%	0%–0%	0%–0%
OFDM2-800	99%–100%	97%–40%	0%–0%	0%–0%	0%–0%	0%–0%
OFDM3-50	100%–100%	100%–100%	49%–0%	0%–0%	4%–3%	0%–0%
OFDM3-100	100%–100%	100%–92%	4%–0%	0%–0%	0%–0%	0%–0%
OFDM3-200	100%–100%	100%–100%	0%–0%	0%–0%	0%–0%	0%–0%
OFDM3-300	100%–100%	100%–86%	0%–0%	0%–0%	0%–0%	0%–0%
OFDM3-400	100%–99%	99%–97%	0%–0%	0%–0%	0%–0%	0%–0%
OFDM3-600	100%–100%	84%–36%	0%–0%	0%–0%	0%–0%	0%–0%
OFDM4-50	99%–98%	100%–100%	3%–3%	0%–0%	0%–0%	0%–0%
OFDM4-100	100%–100%	100%–100%	14%–0%	0%–0%	0%–0%	0%–0%
OFDM4-150	100%–99%	100%–100%	0%–0%	0%–0%	0%–0%	0%–0%
OFDM4-200	100%–99%	99%–95%	0%–0%	0%–0%	0%–0%	0%–0%
OFDM4-300	89%–99%	44%–75%	0%–0%	0%–0%	0%–0%	0%–0%
OQPSK-6.25	100%–100%	100%–100%	100%–96%	44%–37%	100%–87%	22%–64%
OQPSK-12.5	100%–100%	100%–100%	94%–97%	91%–31%	90%–90%	91%–35%
OQPSK-25	100%–100%	100%–100%	100%–100%	47%–34%	92%–100%	4%–14%
OQPSK-50	100%–100%	100%–100%	100%–100%	56%–13%	100%–99%	0%–34%

**Table 9:** Urban Canyon. PDR for packets of 127 B and 2047 B for each RX node, for node locations from 406 m to 585 m. Up to 520 m, all radio settings present a PDR over 50%. PDR of high data rates decay at 540 m.

PHY Alias	RX at 406 m PDR 127 B–2047 B	RX at 512 m PDR 127 B–2047 B	RX at 520 m PDR 127 B–2047 B	RX at 540 m PDR 127 B–2047 B	RX at 570 m PDR 127 B–2047 B	RX at 585 m PDR 127 B–2047 B
2FSK-50	100%–68%	100%–100%	100%–98%	95%–84%	7%–0%	92%–30%
2FSK-100	100%–100%	100%–98%	100%–93%	99%–95%	41%–0%	89%–21%
4FSK-200	94%–100%	100%–99%	93%–99%	98%–87%	0%–0%	0%–0%
2FSK-FEC-50	88%–100%	98%–97%	100%–100%	100%–93%	92%–84%	91%–86%
2FSK-FEC-100	100%–100%	100%–100%	100%–99%	100%–96%	91%–69%	98%–78%
4FSK-FEC-200	100%–100%	100%–99%	100%–100%	100%–95%	42%–26%	78%–39%
OFDM1-100	96%–99%	83%–89%	97%–85%	96%–80%	0%–0%	7%–0%
OFDM1-200	96%–99%	98%–92%	100%–84%	99%–83%	0%–0%	0%–0%
OFDM1-400	90%–90%	36%–61%	99%–94%	97%–91%	0%–0%	0%–0%
OFDM1-800	43%–87%	68%–68%	98%–98%	97%–79%	0%–0%	0%–0%
OFDM2-50	72%–58%	97%–76%	97%–86%	97%–86%	1%–1%	6%–6%
OFDM2-100	17%–59%	97%–88%	99%–93%	97%–86%	0%–0%	58%–4%
OFDM2-200	72%–61%	84%–93%	98%–93%	95%–76%	0%–0%	0%–0%
OFDM2-400	41%–57%	40%–42%	99%–95%	91%–92%	0%–0%	0%–0%
OFDM2-600	98%–98%	29%–0%	98%–97%	97%–88%	0%–0%	0%–0%
OFDM2-800	88%–96%	99%–94%	97%–90%	84%–11%	0%–0%	0%–0%
OFDM3-50	97%–92%	97%–85%	100%–84%	97%–75%	23%–1%	56%–8%
OFDM3-100	95%–90%	82%–90%	98%–93%	99%–93%	2%–0%	10%–0%
OFDM3-200	100%–99%	99%–84%	99%–96%	99%–92%	0%–0%	1%–0%
OFDM3-300	100%–89%	98%–86%	100%–93%	98%–84%	0%–0%	0%–0%
OFDM3-400	99%–89%	99%–88%	89%–64%	86%–42%	0%–0%	0%–0%
OFDM3-600	100%–82%	98%–89%	90%–58%	21%–0%	0%–0%	0%–0%
OFDM4-50	100%–100%	99%–100%	100%–99%	99%–91%	9%–0%	33%–8%
OFDM4-100	100%–100%	98%–98%	99%–100%	97%–96%	0%–0%	33%–0%
OFDM4-150	99%–80%	98%–100%	94%–98%	95%–91%	0%–0%	31%–0%
OFDM4-200	100%–88%	99%–86%	100%–88%	93%–22%	0%–0%	0%–0%
OFDM4-300	99%–99%	100%–92%	88%–72%	60%–31%	0%–0%	0%–0%
OQPSK-6.25	94%–85%	91%–49%	97%–55%	97%–81%	60%–12%	89%–7%
OQPSK-12.5	93%–86%	86%–59%	96%–56%	97%–56%	73%–4%	82%–12%
OQPSK-25	95%–63%	91%–61%	96%–75%	95%–61%	23%–6%	57%–14%
OQPSK-50	91%–74%	90%–76%	99%–86%	98%–84%	41%–19%	67%–48%

**Table 10:** Urban Canyon. PDR for packets of 127 B and 2047 B for each RX node for node locations from 602 m to 942 m The limit of the radio link is at 779 m.

PHY Alias	RX at 602 m PDR 127 B–2047 B	RX at 685 m PDR 127 B–2047 B	RX at 700 m PDR 127 B–2047 B	RX at 742 m PDR 127 B–2047 B	RX at 779 m PDR 127 B–2047 B	RX at 942 m PDR 127 B–2047 B
2FSK-50	0%–0%	99%–94%	2%–0%	0%–0%	42%–0%	0%–0%
2FSK-100	0%–0%	40%–0%	0%–0%	0%–0%	0%–0%	0%–0%
4FSK-200	0%–0%	0%–0%	0%–0%	0%–0%	0%–0%	0%–0%
2FSK-FEC-50	15%–3%	100%–80%	100%–88%	0%–0%	66%–55%	0%–0%
2FSK-FEC-100	8%–0%	100%–46%	38%–4%	0%–0%	17%–0%	0%–0%
4FSK-FEC-200	0%–0%	73%–24%	0%–0%	0%–0%	0%–0%	0%–0%
OFDM1-100	0%–0%	79%–53%	0%–0%	0%–0%	0%–0%	0%–0%
OFDM1-200	0%–0%	55%–1%	0%–0%	0%–0%	0%–0%	0%–0%
OFDM1-400	0%–0%	0%–0%	0%–0%	0%–0%	0%–0%	0%–0%
OFDM1-800	0%–0%	0%–0%	0%–0%	0%–0%	0%–0%	0%–0%
OFDM2-50	0%–0%	90%–54%	51%–2%	0%–0%	2%–0%	0%–0%
OFDM2-100	0%–0%	89%–69%	37%–0%	0%–0%	0%–0%	0%–0%
OFDM2-200	0%–0%	44%–15%	0%–0%	0%–0%	0%–0%	0%–0%
OFDM2-400	0%–0%	11%–0%	0%–0%	0%–0%	0%–0%	0%–0%
OFDM2-600	0%–0%	0%–0%	0%–0%	0%–0%	0%–0%	0%–0%
OFDM2-800	0%–0%	0%–0%	0%–0%	0%–0%	0%–0%	0%–0%
OFDM3-50	0%–0%	97%–94%	0%–0%	0%–0%	12%–1%	0%–0%
OFDM3-100	0%–0%	85%–51%	0%–0%	0%–0%	0%–0%	0%–0%
OFDM3-200	0%–0%	96%–14%	0%–0%	0%–0%	0%–0%	0%–0%
OFDM3-300	0%–0%	16%–0%	0%–0%	0%–0%	0%–0%	0%–0%
OFDM3-400	0%–0%	0%–0%	0%–0%	0%–0%	0%–0%	0%–0%
OFDM3-600	0%–0%	0%–0%	0%–0%	0%–0%	0%–0%	0%–0%
OFDM4-50	0%–0%	93%–9%	0%–0%	0%–0%	0%–0%	0%–0%
OFDM4-100	0%–0%	6%–1%	0%–0%	0%–0%	0%–0%	0%–0%
OFDM4-150	0%–0%	11%–0%	0%–0%	0%–0%	0%–0%	0%–0%
OFDM4-200	0%–0%	0%–0%	0%–0%	0%–0%	0%–0%	0%–0%
OFDM4-300	0%–0%	0%–0%	0%–0%	0%–0%	0%–0%	0%–0%
OQPSK-6.25	36%–0%	88%–53%	99%–79%	15%–0%	86%–16%	29%–1%
OQPSK-12.5	27%–0%	100%–79%	97%–87%	0%–0%	72%–38%	32%–0%
OQPSK-25	8%–1%	98%–66%	92%–64%	0%–0%	73%–18%	0%–0%
OQPSK-50	0%–1%	98%–73%	94%–76%	0%–0%	89%–48%	0%–0%

**Table 11:** AMI. PDR for packets of 127 B and 2047 B long. Even without LoS, high data rates can be achieved up to 215 m with the maximum packet length. At 350 m and 400 m, the PDR decays due to the multiple buildings between TX and RX nodes.

PHY Alias	RX at 126 m PDR 127 B–2047 B	RX at 180 m PDR 127 B–2047 B	RX at 210 m PDR 127 B–2047 B	RX at 215 m PDR 127 B–2047 B	RX at 350 m PDR 127 B–2047 B	RX at 400 m PDR 127 B–2047 B
2FSK-50	100%–100%	100%–100%	100%–100%	100%–100%	0%–0%	0%–0%
2FSK-100	100%–100%	0%–0%	100%–100%	100%–100%	0%–0%	0%–0%
4FSK-200	94%–98%	0%–1%	97%–100%	97%–100%	0%–0%	0%–0%
2FSK-FEC-50	83%–92%	100%–100%	100%–100%	100%–100%	3%–5%	79%–17%
2FSK-FEC-100	100%–100%	100%–73%	100%–100%	100%–100%	0%–0%	0%–0%
4FSK-FEC-200	100%–100%	43%–0%	100%–100%	100%–100%	0%–0%	0%–0%
OFDM1-100	100%–100%	100%–100%	100%–99%	100%–100%	0%–0%	0%–0%
OFDM1-200	100%–99%	100%–100%	93%–100%	100%–98%	0%–0%	0%–0%
OFDM1-400	100%–94%	100%–100%	100%–100%	100%–100%	0%–0%	0%–0%
OFDM1-800	31%–5%	100%–100%	100%–100%	100%–100%	0%–0%	0%–0%
OFDM2-50	84%–100%	100%–100%	99%–99%	99%–83%	0%–0%	0%–0%
OFDM2-100	100%–99%	100%–100%	100%–100%	100%–100%	0%–0%	0%–0%
OFDM2-200	99%–99%	100%–99%	100%–100%	100%–100%	0%–0%	0%–0%
OFDM2-400	100%–99%	100%–100%	100%–100%	100%–100%	0%–0%	0%–0%
OFDM2-600	97%–95%	99%–100%	100%–100%	99%–100%	0%–0%	0%–0%
OFDM2-800	100%–100%	99%–100%	100%–100%	100%–100%	0%–0%	0%–0%
OFDM3-50	100%–100%	100%–100%	100%–98%	100%–100%	0%–0%	0%–0%
OFDM3-100	100%–91%	100%–100%	100%–100%	100%–100%	0%–0%	0%–0%
OFDM3-200	100%–100%	29%–84%	100%–100%	100%–95%	0%–0%	0%–0%
OFDM3-300	100%–72%	100%–99%	100%–100%	100%–87%	0%–0%	0%–0%
OFDM3-400	100%–99%	100%–100%	100%–100%	100%–100%	0%–0%	0%–0%
OFDM3-600	99%–91%	99%–93%	87%–76%	97%–94%	0%–0%	0%–0%
OFDM4-50	100%–100%	99%–100%	100%–100%	100%–98%	0%–0%	0%–0%
OFDM4-100	100%–100%	99%–99%	99%–100%	99%–99%	0%–0%	0%–0%
OFDM4-150	100%–99%	100%–97%	100%–100%	100%–98%	0%–0%	0%–0%
OFDM4-200	99%–100%	100%–98%	100%–100%	100%–99%	0%–0%	0%–0%
OFDM4-300	100%–99%	99%–99%	100%–100%	97%–99%	0%–0%	0%–0%
OQPSK-6.25	100%–96%	100%–100%	100%–100%	100%–100%	97%–10%	76%–24%
OQPSK-12.5	100%–100%	100%–100%	100%–100%	100%–100%	0%–0%	98%–31%
OQPSK-25	100%–100%	100%–100%	100%–100%	100%–100%	3%–0%	98%–68%
OQPSK-50	100%–100%	100%–100%	100%–100%	100%–100%	0%–0%	98%–78%
